# Engineering biomimetic zonal properties in fibre-reinforced hydrogels for functional cartilage tissue engineering *in vitro*

**DOI:** 10.1016/j.mtbio.2025.102550

**Published:** 2025-11-11

**Authors:** Stephen Pahoff, Angus Weekes, Heta Mertano, Johannes J. Braig, Michael W.M. Jones, Anton Maksimenko, Janne T.A. Mäkelä, Petri Tanska, Rami K. Korhonen, Juha Töyräs, Dietmar W. Hutmacher, Travis J. Klein, Christoph Meinert

**Affiliations:** a Australian Research Council (ARC) Centre in Additive Biomanufacturing, Queensland University of Technology (QUT), Brisbane, QLD, Australia; b School of Mechanical, Medical and Process Engineering, Faculty of Engineering, Queensland University of Technology (QUT), Brisbane, QLD, Australia; c Centre for Biomedical Technologies, Queensland University of Technology (QUT), Brisbane, QLD, Australia; d Max Planck Queensland Centre (MPQC), Queensland University of Technology (QUT), Brisbane, QLD, Australia; e Department of Technical Physics, University of Eastern Finland, Kuopio, Finland; f Diagnostic Imaging Center, Kuopio University Hospital, Wellbeing Services County of North Savo, Kuopio, Finland; g Department of Functional Materials in Medicine and Dentistry, Institute of Functional Materials and Biofabrication and Bavarian Polymer Institute, University of Würzburg, Würzburg, Germany; h Central Analytical Research Facility (CARF), Queensland University of Technology (QUT), Brisbane, Queensland, Australia; i School of Chemistry and Physics, Queensland University of Technology (QUT), Brisbane, QLD, Australia; j Centre of Materials Science, Queensland University of Technology (QUT), Brisbane, QLD, Australia; k Australian Synchrotron, ANSTO, Clayton, Victoria, Australia; l School of Electrical Engineering and Computer Science, The University of Queensland, Brisbane, QLD, Australia; m Science Service Center, Kuopio University Hospital, Wellbeing Services County of North Savo, Kuopio, Finland; n Gelomics Pty Ltd., Brisbane, QLD, Australia

## Abstract

The functional regeneration of human articular cartilage is hampered by a lack of biomaterials and tissue engineering strategies that adequately capture the physiological depth-dependent compression properties of native tissue. Here, we demonstrate that photocrosslinkable gelatin-hyaluronic acid hydrogels reinforced with multiphasic polycaprolactone microfibre scaffolds form biomimetic soft network composites that serve as *in vitro* models mimicking the compressive and depth-dependent deformation characteristics of human cartilage. Mono- and multi-phasic gradient scaffolds with fibre spacings of 200, 400, and 800 μm were manufactured using melt electrowriting and embedded in the photocrosslinkable hydrogel system. Mechanical testing combined with finite element analysis revealed how defined microfibre architecture is altered and influences compressive moduli and interstitial fluid load support to mimic the loading response of native articular cartilage in our *in vitro* model. Digital image and volume correlations demonstrated depth-dependent strain fields in the fibre-reinforced constructs in response to compression, demonstrating biomimetic depth-dependent behaviour. Lastly, we demonstrate that these fibre-reinforced hydrogels support high cell viability, chondrogenic redifferentiation and hyaline-like tissue formation by expanded human articular chondrocytes *in vitro*. Together, this study demonstrates that, *in vitro*, these hydrogels reinforced with gradient scaffolds successfully recapitulate key biomechanical traits of native articular cartilage, toward the development of improved models for functional cartilage tissue engineering.

## Graphical abstract

**Figure:**
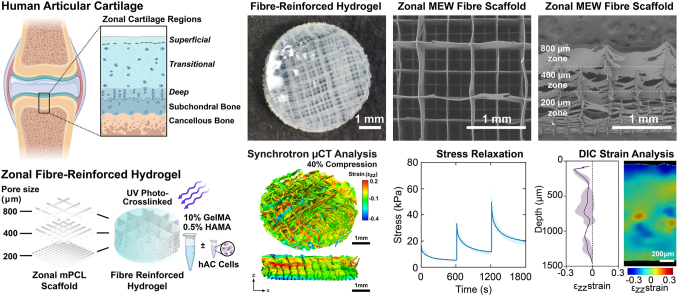

## 1 Introduction

Matrix-assisted autologous chondrocyte implantation (MACI) is a tissue engineering strategy for articular cartilage defect repair that relies on the implantation of a three-dimensional (3D) collagen scaffold seeded with autologous chondrocytes [1]. However, post-operative assessment of the resulting repair tissue demonstrates a significant predisposition towards mechanically inferior fibrocartilage that lacks the native zonal stratification required for the tissue's physiological mechanical function and durability [2]. Owing to their customisable characteristics and ability to mimic native tissues, hydrogels are widely regarded as prime candidates for the next generation of MACI scaffolds that may overcome current limitations. Among the many varieties investigated, photocrosslinkable gelatin methacryloyl (GelMA)/hyaluronic acid methacrylate (HAMA) [[3], [4], [5]] hydrogels have been utilised extensively in functional articular cartilage tissue engineering due to their tuneable mechanical properties, cytocompatibility, cell binding and enzymatic degradation motifs, and superior ability to support hyaline cartilage regeneration. However, conventional GelMA-based hydrogels possess compressive moduli that are typically multiple orders of magnitude lower than that of articular cartilage, lack the toughness required to support intermittent cyclic and high-force loading, and fail to recapitulate the depth-dependent mechanical behaviour of native tissue, thereby limiting their usefulness in musculoskeletal tissue engineering applications [6,7].

These mechanical limitations have prompted various modifications to hydrogel systems such as double network [8] and self-healing hydrogels [9]. Our group has developed an alternative approach that relies on reinforcing intrinsically soft hydrogel structures with highly organised microfibre scaffolds [10] produced using melt electrowriting (MEW) technology [11,12]. Remarkably, microfibre reinforced hydrogels exhibit dramatically increased stiffness and enable the precise tailoring of mechanical properties that are critical to the function of musculoskeletal tissues [13] while supporting *in vitro* hyaline-like neocartilage development [11]. To better mimic natural tissue organisation, our group has recently developed gradient scaffolds with discrete zones of defined scaffold porosities that, while not full replicating the very high bulk mechanics of native tissue, when used to reinforce GelMA/HAMA hydrogels, functionally recapitulate the depth-dependent compression patterns of native cartilage tissue [14]. Whilst the utilisation of these gradient composites is appealing for cartilage repair, it remains unclear whether they adequately recapitulate the depth-dependent strain mechanics of native articular cartilage under loading conditions.

X-ray micro-tomography (μCT) is occasionally used to image soft composites such as fibre-reinforced hydrogels under loading. However, the spatial and temporal resolution is often limited in hydrogels due to imaging artefacts generated by stress relaxation during long scan times [[15], [16], [17]]. Overcoming these limitations, synchrotron x-ray μCT (sCT) is a high flux imaging technique which is capable of rapidly capturing large 3D volumes with greater spatial resolution [18]. sCT imaging has been used to gain morphological insights into musculoskeletal tissues such as bone [19], cartilage [20] and muscle [21,22]. Recently, Madi et al. [18] utilised *in situ* sCT imaging of mouse knee joints at different levels of physiological mechanical loading. The volumetric datasets were analysed using digital volume correlation (DVC) analysis, a technique that determines three-dimensional displacements and strains. Ultra-high resolution sCT imaging (effective pixel size of 1.6 μm) in combination with DVC analysis was able to resolve displacements and strains within the calcified cartilage component of the mouse knee. Whilst a promising technique, the combination of sCT and DVC for biomedical applications has not been widely explored, with investigations primarily focused on biomaterial-based bone implants [23,24]. To our knowledge, these techniques have not been applied to fibre-reinforced hydrogels for articular cartilage tissue engineering applications.

Characterisation of the underlying mechanics of fibre-reinforced hydrogels have thus far been restricted to measuring bulk mechanical properties or utilising simulations [[12], [13], [14], [15],^25^]. Detailed constituent-specific mechanical material properties can, however, be obtained by combining sophisticated material models and finite element (FE) modelling. Fibre-reinforced hydrogels have been reported to correspond to the overall mechanical behaviour of native articular cartilage [12,14] which is well-captured by fibril-reinforced poroelastic (FRPE) material models consisting of a fibrillar and a fluid-filled poroelastic matrix, respectively [[26], [27], [28], [29], [30]]. FRPE models may provide valuable insights into the remarkable reinforcement effect observed in microfibre hydrogel composites. Thus, through combination of these analytical techniques and computational processes, this study aimed to characterise the compressive properties of monophasic and gradient mPCL microfibre-reinforced hydrogels and investigate the depth-dependent strain behaviour of the hydrogel phase and the mPCL scaffold phase of reinforced hydrogel constructs in response to compressive loading.

## 2 Methods

### 2.1 mPCL scaffold fabrication

Microfibre scaffolds with an alternating offset angle of 0° and 90° were fabricated as per Bas et al.*,* [14] with medical grade polycaprolactone (mPCL) (Purasorb PC12, Corbion, Netherlands) using a custom-built MEW device [[31], [32], [33]]. Scaffolds were either printed with a ‘monophasic’ structure (uniform fibre spacing throughout the scaffold height, with 200, 400 or 800 μm pore size, respectively; scaffold height: 1.39 ± 0.02 mm, 1.43 ± 0.01 mm, 1.44 ± 0.03 mm, respectively) or gradient scaffolds that combined all pore sizes (200, 400 or 800 μm) in a stacked gradient pattern, with the 200 μm region printed initially and the 400 and 800 μm regions printed subsequently above the preceding layer (total scaffold height: 1.36 ± 0.02 mm; 800 μm zone height: 0.45 ± 0.09 mm; 400 μm zone height: 0.43 ± 0.04 mm; 200 μm zone height: 0.46 ± 0.01 mm). Scanning electron microscopy (SEM) (TM3000, Hitachi, Japan) analysis of the scaffolds was used to determine fibre diameters (n = 9), scaffold fibre spacing (n = 10) and scaffold height (n = 6). Measurements were quantified using ImageJ software (National Instruments, USA).

### 2.2 Composite construct preparation

mPCL scaffolds were biopsy punched (5 mm diameter) and plasma-treated with oxygen and argon in a 1:1 vol ratio in a plasma cleaner (Harrick Plasma, USA). Scaffolds were inserted into a custom-designed polytetrafluoroethylene (PTFE) casting mould (5 mm diameter x 1.5 mm depth), with gradient scaffolds placed with the 800 μm pore size side facing up. Throughout, gelatin methacryloyl (GelMA; bovine bone, type B, 80 % degree of functionalisation) and hyaluronic acid methacrylate (HAMA, M_W_ = 0.86 MDa) were used (both Gelomics Pty Ltd., Australia). A 10 % w/v GelMA/0.5 % w/v HAMA hydrogel precursor solution with 0.25 % w/v Irgacure 2959 (IC2959; 1-[4-(2-hydroxyethoxy)-phenyl]-2-hydroxy-2-methyl-1-propanone; BASF, Germany) was heated to 37 °C and pipetted into the moulds containing the mPCL scaffolds [11]. Hydrogel/mPCL composites were photocrosslinked for 15 min at 365 nm in a CL-1000 crosslinker (UVP, USA). Constructs were incubated overnight at 37 °C in PBS to allow for hydrogel swelling before further testing.

### 2.3 Mechanical testing and elastic analysis

Prior to mechanical testing, calibrated light microscope images of the reinforced hydrogel constructs were obtained and analysed with ImageJ to determine construct surface area for load normalisation. Unconfined compression testing was performed using an Instron 5567 (Instron, USA) equipped with a 500 N load cell following previously published methods [34] and mechanical parameters were determined using a published open-source algorithm [35]. Stepwise stress-relaxation tests were conducted as previously described [36] to determine the equilibrium properties of the hydrogel constructs using an independent set of samples. Briefly, constructs were compressed from 0 to 15 % strain in 5 % increments at a displacement rate of 0.01 mm/s, with a 10-min relaxation period following each step. The equilibrium modulus was calculated from the slope of the equilibrium stress vs. strain for each step (5 %, 10 % and 15 %). For unreinforced hydrogel constructs, an additional compression step of 5 % (to 20 % total strain) with a 10-min relaxation period was conducted, to provide more information on the possible strain-dependent behaviour of the unreinforced constructs.

### 2.4 Inverse finite element analysis to obtain unknown material properties

Stepwise stress-relaxation tests were replicated using finite element (FE) modelling with Abaqus (v2020, Dassault Systémes, USA). Monophasic and gradient mPCL reinforced hydrogel constructs, as well as unreinforced hydrogel constructs, were modelled in an axisymmetric geometry using axisymmetric linear pore pressure continuum elements (type CAX4P). The model geometry was sample-specific (diameter of 5 mm; height determined from the mechanical testing data), and the mesh density was chosen based on convergence analysis (375 or 400 elements, depending on the sample height). Reinforced hydrogel constructs were modelled as fibril-reinforced poroelastic (FRPE) materials consisting of a fibrillar matrix and a poroelastic non-fibrillar matrix filled with fluid [37].

The unreinforced hydrogel constructs were modelled as a poroviscoelastic material, consisting of a fluid and porous hyperviscoelastic solid matrices [38]. Optimisation of the material parameters was carried out by fitting the simulated axial loading to the experimentally measured loading data. The optimised material parameters were initial fibril network modulus, strain-dependent fibril network modulus, non-fibrillar matrix modulus, initial permeability and strain-dependent permeability factor ($E_{f}^{0},E_{f}^{\varepsilon },E_{\text{nf}},k_{0}$ and M, respectively) for the mPCL-reinforced hydrogel constructs and solid matrix modulus, initial permeability, Prony constant corresponding to dimensionless shear modulus and Prony series characteristic relaxation time ($E_{s},k_{0},{\bar{g}}_{1}$ and $\tau_{1}$, respectively) for unreinforced hydrogel constructs. Interstitial fluid load support (IFLS) was calculated by dividing the pore pressure by the contact pressure. Detailed descriptions of the material models and FE analysis can be found in the Supplementary Data.

### 2.5 Digital image correlation (DIC) of compressed reinforced hydrogels

**Fig. 1:**
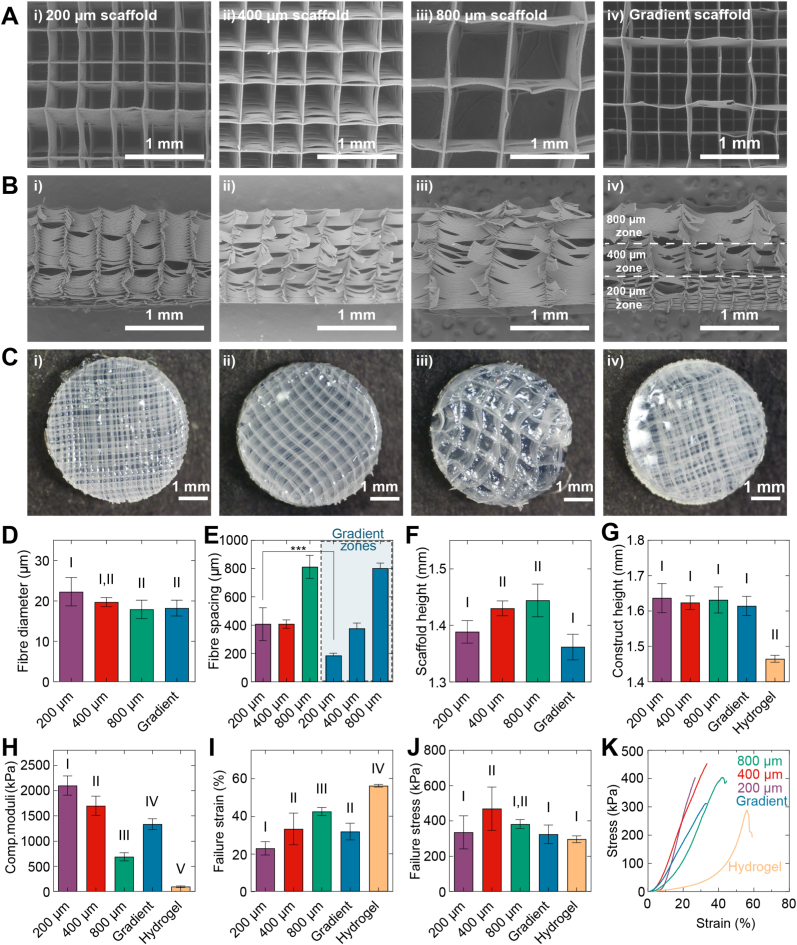
**Characterisation of melt-electrowritten (MEW) mPCL scaffold properties and compression properties of mPCL scaffold-reinforced GelMA/HAMA hydrogel composite constructs.** (A) SEM images of 0–90° lay-down pattern melt-electrowritten microfibre scaffolds with 200, 400 and 800 μm fibre spacings and gradient scaffold with three separate fibre spacings (from top to bottom: 800, 400 and 200 μm) (40x magnification). (B) Cross-sectional SEM images of mPCL scaffolds (40x magnification). (C) Stereomicroscope images showing gross morphology of GelMA/HAMA hydrogels reinforced with MEW mPCL scaffolds. (D) Fibre diameter measured from SEM images. (E) Fibre spacing, (F) mPCL scaffold height and (G) the total height of the reinforced hydrogel construct. (H) Compressive modulus, (I) failure strain and (J) failure stress of reinforced hydrogel constructs. (K) Representative stress-strain curves of reinforced and unreinforced hydrogels during unconfined compression. Groups that do not share a Roman numeral are statistically different (one-way ANOVA, p < 0.05) (mean ± SD).

As previously published by us [39,40], digital image correlation (DIC) was used to determine the deformation and strain within the hydrogel matrix under loading. mPCL-reinforced hydrogel constructs containing Fluoresbrite® fluorescent microspheres (Polysciences, Taiwan; 1 × 10^7^/mL) were prepared as above and allowed to swell overnight in PBS at 37 °C. Hydrogels were halved to expose a cross section and then loaded into a custom-designed, microscope-mounted loading system equipped with a linear actuator (Zaber Technologies, Canada) and a custom indenter [39,40]. For imaging, the samples were orientated as manufactured, as shown in Fig. 1B; where, for the gradient composites, the 200 μm region was located at the bottom, the 400 μm region in the middle, and the 800 μm region at the top. Samples were compressed at a rate of 0.01 mm/s until 20 % compressive strain was reached. At each compression step, an image was taken using a SP5 Confocal microscope (Leica Microsystems, Germany). Hydrogel strains and displacements were determined using Ncorr digital image correlation (DIC) open-source software [41] (v1.2.2; subset radius: 35, subset spacing: 5).

### 2.6 Synchrotron imaging (sCT)

Synchrotron x-ray μCT was performed to investigate the deformation of mPCL microfibre scaffolds in reinforced constructs using the Imaging and Medical Beamline [42] at the Australian Synchrotron (Australia's Nuclear Science and Technology Organisation) (Fig. S1, Supplementary Data). Prior to imaging, constructs were incubated in Lugol's iodine solution at 37 °C for ∼1 h to enhance fibre contrast. Phase-contrast imaging was conducted using a monochromatic 25 keV beam from a 3T multipole wiggler with the sample placed approximately 135 m from the source. A lens-coupled 5.5-megapixel CMOS detector with a pixel size of 6.3 μm was placed approximately 6 m downstream of the sample. During imaging, samples were compressed with a polycarbonate plunger connected to a micro-linear actuator (microstep size <50 nm) with a unidirectional accuracy of 15 μm (Fig. S1, Supplementary Data) [43]. The actuator was connected to a 50 N load cell that provided real-time force feedback during uniaxial sample compression. Actuator position and load cell readout was controlled remotely during imaging.

### 2.7 sCT image processing and reconstruction

32-bit sCT tomograms were converted to 8-bit, brightness adjusted and cropped in ImageJ prior to 3D reconstruction and Digital Volume Correlation (DVC) analysis (Fig. S1, Supplementary Data). Edited images were then imported to Amira (2019.4) software and median filtered (kernel size 3) to remove noise and segmented using the interactive thresholding tool. 3D reconstructions were generated using the generate surface function with an unconfined smoothing extent of 3.5 (Fig. S1, Supplementary Data). Morphological properties of mPCL scaffolds were quantified from 3D reconstructions at each compression step (n = 2 per construct type) to determine scaffold pore width (n = 10 measurements per strain level), height (n = 5 measurements per strain level) and total scaffold width (n = 5 measurements per strain level) in response to loading. To assess the depth-dependent response to loading in gradient scaffolds, pore size and scaffold height were determined for each fibre spacing region (*i.e.,* 200, 400 and 800 μm).

### 2.8 Digital volume correlation (DVC) of sCT reconstructions

Digital volume correlation (DVC) analysis was conducted using Amira (2019.4) software to measure the stepwise deformation characteristics of mPCL microfibres in hydrogel composites compressed during synchrotron imaging [44]. Edited 8-bit image non-deformed and deformed volumes were co-registered to remove rigid body motion (translation and rotation) [18]. A subset-based (local) DVC approach was first used to define an initial displacement field which was subsequently used to initialize a global DVC approach using a tetrahedral mesh (200 μm voxel size). Following DVC processing, strain fields were superimposed as a colour wash on the scaffold 3D surface. Strain magnitude was measured using the line probe tool (n = 3 measurements per strain type at each compression step).

### 2.9 Scaffold fibre properties from sCT DVC reconstructions

To determine the effect of compression on mPCL fibre orientation, each tomogram slice of the scanned constructs was analysed using OrientationJ in ImageJ software. Fibre orientation histograms were generated for each slice, then averaged across the scaffold height. The degree of fibre straightness was also measured to determine depth-dependent fibre behaviour under axial loading. Fibre straightness (*F*_*s*_) was determined by the ratio of the distance between the endpoints of a fibre (*L*_*0*_) and actual fibre length (*L*_*f*_) (Equation (1)). When *F*_*s*_ = 1, fibres are perfectly straight, with fibres becoming wavier towards *F*_*s*_ = 0. Fibre straightness was measured in three separate regions (top, middle, bottom) at each strain level of sCT 3D reconstructions (n = 2 per construct type, n = 5 fibres per region).

(1) $$F_{s}=\frac{L_{0}}{L_{f}}$$

### 2.10 Cell isolation, expansion, and encapsulation

Chondrocytes were isolated from macroscopically normal full thickness cartilage of the lateral and medial femoral condyles, as described elsewhere [45]. Cells were expanded in low-D-glucose chondrocyte basal medium (Dulbecco's modified Eagle's medium (DMEM)) supplemented with 2 mM GlutaMAX™, 10 mM 4-(2-hydroxyethyl)-1-piperazineethanesulfonic acid (HEPES), 0.1 mM MEM non-essential amino acid solution, 50 μg/mL penicillin/streptomycin, 0.5 μg/mL amphotericin B (Fungizone®) (all Invitrogen, USA), 0.4 mM L-proline, 0.1 mM L-ascorbic acid (both Sigma Aldrich, St. Louis, MO) and 10 % fetal bovine serum (FBS) (Hyclone, USA). Passage 1 chondrocytes were encapsulated in multiphasic mPCL microfibre-reinforced GelMA/HAMA constructs (prepared as above) at a density of 1 × 10^7^ cell/ml and cultured at 37 °C in differentiation media composed of serum-free high-D-glucose basal chondrocyte medium (composition same as expansion media) supplemented with insulin-transferrin-selenium (ITS-G) (100x dilution), 1.25 mg/mL bovine serum albumin, 0.1 μM dexamethasone (all Sigma Aldrich) and 10 ng/mL human recombinant transforming growth factor beta-3 (TGF-β3) (GroPep, Australia) for 42 days.

### 2.11 Biochemical analysis

For the biochemical analysis of GAG and DNA content, hydrogel constructs were weighed and enzymatically digested overnight in phosphate-buffered EDTA (pH 7.1) containing 1 mg/mL hyaluronidase (Sigma) at 37 °C followed by addition of 0.5 mg/mL Proteinase K (Invitrogen) and digesting overnight at 56 °C. GAG content of digested samples was determined with a dimethylmethylene blue (DMMB) assay (pH 1.5). The absorbances of construct digests at 525 and 595 nm were measured with a CLARIOstar microplate reader (BMG Labtech, Australia) and compared to a chondroitin sulfate (Sigma) quadratic standard curve. DNA content was determined using the Quant-iT™ PicoGreen® dsDNA quantification assay (Invitrogen) on construct digests following the manufacturer's instructions (2 donors, 3–4 constructs per donor).

### 2.12 Immunofluorescence analysis

At day 14 and 42 of culture, hydrogel constructs were fixed with 4 % (wt/vol) paraformaldehyde for 30 min at RT and stored in PBS at 4 °C (1 donor, 1 construct per experimental condition). Samples were permeabilised/blocked with 0.1 % (v/v) TritonX in PBS containing 2 % (v/v) goat serum overnight at 4 °C. Samples were then incubated with 0.1 % hyaluronidase (Sigma) in PBS at 37 °C for 2 h for antigen retrieval. Primary antibodies for collagen type I (ab34710, Abcam, 1:300 in PBS with 2 % goat serum) or collagen type II (II-II6B3, Developmental Studies Hybridoma Bank (DSHB), USA; 1:100 dilution in PBS with 2 % goat serum) were applied overnight at 4 °C, respectively. Secondary antibodies were diluted 1:150 in PBS with 2 % goat serum (all AlexaFluor® 488-labelled; all Jackson ImmunoResearch, USA) and 5 μg/mL 6-diamidino-2-phenylindole (DAPI) (Invitrogen) were applied overnight at 4 °C. Samples were imaged with a Leica SP5 confocal laser scanning microscope (Leica) and maximum intensity projections were generated.

### 2.13 Statistical analysis

Statistical analysis was performed using GraphPad Prism (GraphPad, USA) and MATLAB (MathWorks, USA). Differences between groups for scaffold fibre and elastic mechanical properties as well as optimised material parameter were assessed using one-way analysis of variance (ANOVA) with Tukey's post-hoc test. For unreinforced hydrogel constructs, Friedman's test with Tukey's post-hoc analysis was used for comparing material parameters between the steps in FE analysis. Two-way ANOVA with Tukey's post-hoc analysis was used for scaffold morphology measurements from 3D volumes and fibre straightness parameter analysis. For fibre orientation analysis, the Kolmogorov*-*Smirnov test was used to compare fibre angle histograms with the uncompressed scaffold with each subsequent compressive strain level. The level of statistical significance was 0.05 for all analyses.

## 3 Results

### 3.1 Fibre characteristics and compression properties of mPCL scaffolds and fibre-reinforced hydrogel constructs

Monophasic and gradient mPCL scaffolds were printed using melt electrowriting (MEW) and subsequently analysed by SEM imaging. All scaffold types were accurately printed with a 0–90° lay-down pattern ordered fibre stacking (Fig. 1A). However, transverse fibres exhibited variable degrees of sagging, particularly in the 800 μm scaffold and 800 μm region of the gradient scaffold (Fig. 1B). Stereomicroscopy showed that the hydrogel solution effectively infiltrated the scaffolds with no visible bubble formation (Fig. 1C). Quantitative image analyses of the SEM data obtained revealed both fibre diameter (19.7 ± 1.1 μm and 17.9 ± 2.3 μm, respectively) and fibre spacing (408.5 ± 30.4 μm and 813 ± 81.1 μm, respectively) were tightly controlled in the 400 and 800 μm scaffold groups (Fig. 1D and E). Gradient scaffolds also exhibited consistent fibre diameter (18.2 ± 2.0 μm) and spacing throughout the scaffold (802.2 ± 36.8 μm, 377.3 ± 38.36 μm and 185.5 ± 16.8 μm for the 800, 400 and 200 μm zones, respectively) as observed in the SEM images (Fig. 1D and E). Although the 200 μm scaffold group exhibited accurate fibre spacing for the first ∼10–15 deposited layers, fibres became progressively merged until fibre spacing increased dramatically (409 ± 116 μm, 103.9 % error compared to expected pore size) (Fig. 1D).

Fibre merging may have also caused the increased fibre diameter and increased standard deviation observed in the 200 μm scaffold group (22.3 ± 3.5 μm) (Fig. 1D). All scaffold types were slightly shorter than the expected height of 1.5 mm (200 μm scaffold: 0.11 mm, 7.41 % error; 400 μm scaffold: 0.07 mm, 4.6 % error; 800 μm scaffold: 0.05 mm, 3.7 % error; gradient scaffold: 0.14 mm, 9.2 %) (Fig. 1F). The addition of the hydrogel component to the scaffolds normalised the overall hydrogel construct heights, with no significant differences noted between groups. Unreinforced hydrogels were significantly shorter by comparison to all reinforced groups following swelling (p < 0.0001) (Fig. 1G).

The compressive modulus of unreinforced GelMA/HAMA hydrogels was 94 ± 16 kPa (Fig. 1H). The addition of mPCL scaffolds to hydrogels significantly increased construct stiffness compared to unreinforced hydrogels with a 22-fold, 18-fold, 7-fold and 14-fold increase in compressive modulus of the 200, 400, 800 μm and gradient scaffold-reinforced groups (2102 ± 191 kPa, 1702 ± 189 kPa, 690 ± 82 kPa and 1335 ± 110 kPa, respectively, all p < 0.001). Importantly, the bulk compressive modulus of gradient scaffold-reinforced gels was well in line with moduli reported for human cartilage (∼1600 kPa) assessed using similar mechanical testing protocols [12]. Notably, while construct compressive moduli inversely correlated with fibre spacing, a positive correlation of fibre spacing and failure properties was observed, with smaller fibre spacing resulting in more brittle constructs (Fig. 1I–K).

Notably, the compressive failure stress was significantly higher in the 400 μm scaffolds (469 ± 123 kPa), with all other groups failing between 296 and 382 kPa (Fig. 1J). Despite compression exceeding the failure points, reinforced constructs remained macroscopically intact and were able to bear load following completion of the mechanical tests, while hydrogels alone fragmented into multiple pieces (not shown).

### 3.2 Mechanical characterisation of stress-relaxation behaviour

**Fig. 2:**
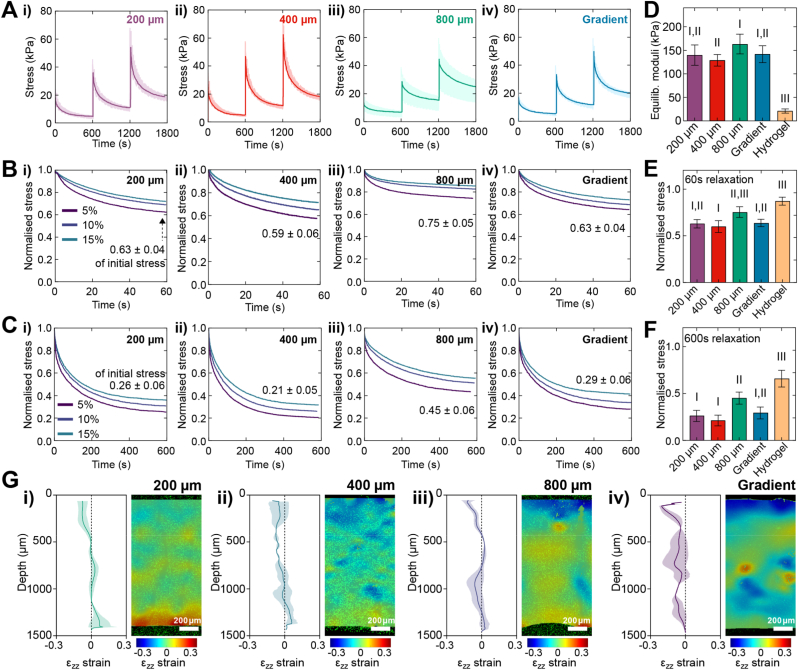
**Mechanical characterisation of stress-relaxation behaviour of mPCL microfibre scaffold-reinforced GelMA/HAMA hydrogels alongside DIC imaging analyses.** (A) Stepwise stress-relaxation curves (mean = dark line; SD = lighter shading, n = 5–7 per construct type. (B) Normalised stress-relaxation curves with 60 s hold time at 5, 10 and 15 % compression steps. (C) Normalised stress-relaxation curves with 600 s hold time at 5, 10 and 15 % compression steps. To quantify the degree of relaxation, the difference between the normalised peak stress and stress at (D) 60 or (E) 600 s following loading is shown. (F) Modulus of reinforced and unreinforced hydrogel constructs at 600 s. (G) Representative DIC images of compressive strain of the hydrogel phases within mPCL microfibre-reinforced constructs under axial compression (20 % compressive strain) with superimposed strain visualisation, where corresponding line graphs represent strain across the depth of the construct (mean = line, standard deviation = lighter shading; *n* = 2 per construct type; scalebar = 200 μm); noting that samples for DIC imaging were orientated as shown in Fig. 1B, where for the gradient group the smaller porosity layer is at the bottom. For all graphs, groups that do not share a Roman numeral are statistically different (mean ± SD, n = 5–7 per construct type).

Fig. 2 depicts the stress-relaxation behaviour of mPCL-reinforced GelMA/HAMA hydrogels during stepwise compression testing. All constructs displayed a viscoelastic response to loading (Fig. 2A–C). The equilibrium moduli of reinforced gels were significantly higher than hydrogels alone with only minor differences between scaffold types (Fig. 2D), suggesting that the observed differences in bulk compressive moduli (Fig. 1H) may be primarily driven by differential fluid pressurisation in reinforced groups with varying scaffold architectures. The speed of relaxation of compressed hydrogels is an important regulator of chondrocyte function, with faster relaxing constructs promoting a greater volume of synthesised matrix [46]. In this study, we normalised the stress-strain curves at different compression levels to determine relaxation at a short (60 s) (Fig. 2B–E) and long timescales (600 s) (Fig. 2C–F) For each timescale, the normalised stress of the gradient group did not significantly differ from that of the 200, 400 or 800 μm groups (Fig. 2E and F). However, the gradient group's normalised stress was significantly reduced compared to the hydrogel only. While the bulk normalised stresses did not differ significantly between reinforced composite groups, the depth-dependent strain profiles differed when analysed via DIC, as described subsequently (Fig. 2G). Additionally, as full experimental relaxation was not achieved, the inverse FE and fibril-reinforced material model was utilised to calculate the equilibrium response of each group with simulated 30-min relaxation time between steps (Table S2, Supplementary Data). Notably, the highest calculated modulus was observed in the 800 μm group, validating that while absolute moduli values decreased further with 30-min relaxation, the trend remained consistent.

### 3.3 DIC image analysis of depth-dependent strain

To investigate the deformation characteristics of unreinforced hydrogel and reinforced composites, we assessed the depth-dependent strain in the hydrogel phase in response to compression through DIC imaging of compressed constructs embedded with fluorescent microparticles (Fig. 2G; Fig. S2, Supplementary Data). DIC imaging revealed hydrogels reinforced with each scaffold type exhibited unique depth-dependent strain characteristics at maximal axial compression (20 % compressive strain) primarily in the axial direction (Fig. 2G). At maximal compression, gradient constructs exhibited the largest local compressive strain *Ɛ*_*zz*_, which was observed primarily in the surface region (20.45 % *Ɛ*_*zz*_) (Fig. 2G (iv)). Similar to the depth-dependent compression patterns known to be present in native cartilage [47], hydrogel compressive strain in the gradient mPCL-microfibre scaffold reinforced composites appeared to progressively decrease with depth (0–500 μm from surface region: 8.9 ± 5.3 % *Ɛ*_*zz*_; 500–1000 μm region: 6 ± 1.9 % *Ɛ*_*zz*_; 1000–1500 μm region: 4.9 ± 2.8 % *Ɛ*_*zz*_) (Fig. 2G (iv)). Whilst the 800 μm monophasic scaffold group showed similar *Ɛ*_*zz*_ behaviour at the surface of the hydrogel phase (12.3 % *Ɛ*_*zz*_), limited *Ɛ*_*zz*_ was observed in the mid to lower regions (Fig. 2G (iii)). In comparison, the 200 and 400 μm scaffold groups were dominated by axial tensile strain in the mid to lower regions of the hydrogel (Fig. 2G (i,ii)).

DIC imaging of constructs under mechanical compression were also analysed in the lateral and shear strain planes (Fig. S2, Supplementary Data). Hydrogel lateral strain *Ɛ*_*xx*_ appeared to loosely correlate with the fibre spacing of the scaffold, with strain increasing or decreasing in an alternating pattern. Larger fibre spacing correlated with increases in *Ɛ*_*xx*_, with both the 400 and 800 μm monophasic groups and 400 and 800 μm regions of the gradient scaffold displaying a large degree of lateral strain. The 200 μm fibre spacing in monophasic and gradient scaffolds did not show a large change in hydrogel lateral strain behaviour. Shear strain (*Ɛ*_*xy*_) within the hydrogel phase was most pronounced in the 800 μm construct group, which experienced the largest increase across the depth of the construct (12.81 %), with a region of shear strain developing in the 1000–1500 μm deep region (Fig. S2, Supplementary Data). Shear strain was visible in the gradient group primarily in the central portion. In contrast, both 200 and 400 μm groups displayed limited shear strain behaviour. FE-material parameters of fibre-reinforced and unreinforced hydrogel constructs.

**Fig. 3:**
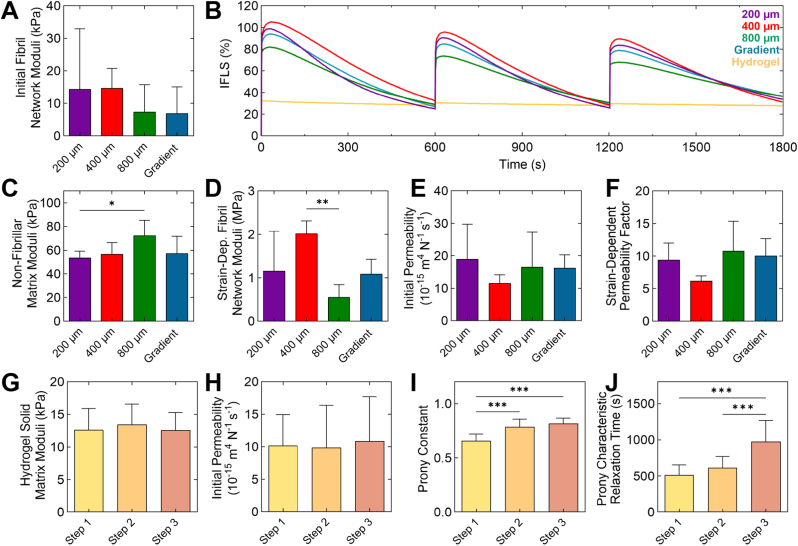
**Material parameters and IFLS of mPCL scaffold-reinforced and unreinforced GelMA/HAMA hydrogels.** (A) Initial fibril network moduli, (B) mean interstitial fluid load support (IFLS) across multiple cycles, (C) non-fibrillar network modulus, (D) strain-dependent fibril network modulus, (E) initial permeability and (F) strain-dependent permeability coefficient of reinforced hydrogel constructs (mean ± SD, n = 5–7 per construct type). (G) Hydrogel solid matrix modulus, (H) initial permeability, (I) Prony constant corresponding to dimensionless shear modulus, and (J) Prony series characteristic relaxation time of unreinforced hydrogel constructs (mean ± SD, n = 11). Stress-relaxation steps 1, 2 and 3 correspond to 10, 15 and 20 % total compression, respectively. Stars indicate a statistically significant difference between groups (one-way ANOVA, ∗p < 0.05, ∗∗p < 0.01 and ∗∗∗p < 0.001).

In computational FE simulations, a fibril-reinforced poroelastic (FRPE) model was able to adequately replicate the experimental stress-relaxation behaviour of fibre-reinforced hydrogel constructs ($R^{2}=0.991\;\pm 0.012$ (mean ± SD)) (Fig. 3; Supplementary Table 1). The interstitial fluid load support (IFLS) is the fraction of total load supported by fluid pressurisation inside biphasic material pores. In cartilage, it contributes to the tissue's remarkable mechanical and tribological properties [48,49]. Peak IFLS was greater for reinforced constructs compared to unreinforced hydrogel constructs, suggesting the presence of reinforcing microfibres increases fluid pressurisation in response to compression. At the beginning of each compression test, most of the load in the reinforced hydrogel constructs was carried by pressurised fluid (approx. 80–100 %). In contrast, in unreinforced hydrogel constructs, only around 30 % of the load was carried by the fluid throughout the compression.

The strain-dependent fibril network modulus – a measure of the fibrils’ ability to stiffen in response to increased tension – was significantly higher in the 200 and 400 μm compared to the 800 μm scaffold group. Trends observed for the non-fibrillar matrix modulus were inversely correlated and increased with scaffold pore size, with a statistically significant difference between the 200 μm and 800 μm groups (p < 0.05). Optimised material parameters for unreinforced constructs were obtained separately for each stress-relaxation step (Fig. 3; Supplementary Table 1). A hyperviscoelastic model was able to mimic the stress-relaxation behaviour of separate steps of the unreinforced hydrogel constructs with a coefficient of determination of $R^{2}=0.988\;\pm 0.005$ (mean ± SD). The Prony constant, corresponding to the dimensionless shear modulus, was found to increase with compressive strain applied. Additionally, the Prony series characteristic relaxation time (*i.e.*, time required to reach stress equilibrium under constant strain) significantly increased with applied compressive strain, suggesting viscous behaviour increases with strain.

### 3.4 Morphological changes to mPCL scaffolds in reinforced constructs under axial loading

**Fig. 4:**
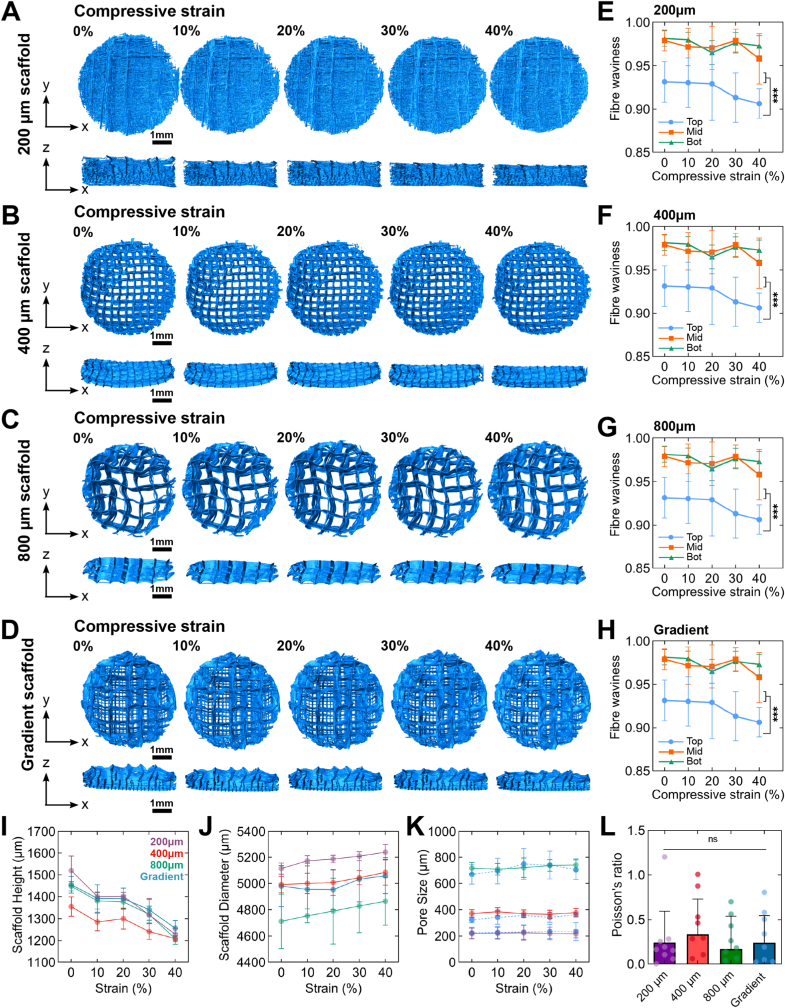
**3D reconstructions and morphological analysis of composite reinforced scaffold constructs from synchrotron x-ray μCT at different axial compression steps.** Reinforced hydrogel constructs were compressed in discrete 10 % compression steps and imaged using during synchrotron x-ray μCT at each step. Constructs were analysed to determine changes in bulk morphological properties. (A–D) 3D reconstructions of (A) 200 μm, (B) 400 μm, (C) 800 μm and (D) gradient fibre-spaced mPCL-reinforced hydrogel constructs at 0 %, 10 %, 20 %, 30 % or 40 % axial compression. (E–H) Quantified fibre straightness (*F*_*s*_) determined by the ratio of the distance between the endpoints of a fibre (*L*_*0*_) and the fibre length (*L*_*f*_). Fibre straightness increases as the straightness coefficient (*F*_*s*_) moves closer to 1.0, measured at three separate regions (top, middle, bottom) of mPCL scaffolds. (mean ± SD; n = 2 per construct type, n = 5 fibres per measured region). (I) Scaffold height, (J) scaffold diameter, (K) scaffold pore size, and (L) calculated Poisson's ratio of mPCL scaffold morphologies within reinforced constructs from 0 to 40 % compressive strain, ascertained from 3D sCT reconstructions (mean ± SD; *n* = 10 measurements). Stars indicate a statistically significant difference between measured layers at 40 % total compressive strain (two-way ANOVA, ∗p < 0.05, ∗∗p < 0.01 and ∗∗∗p < 0.001).

To analyse how the mPCL fibre scaffold component of reinforced hydrogels behaved under axial compression, we analysed the constructs at discrete axial compression steps using synchrotron computed tomography (sCT) and reconstructed these images into 3D models (Fig. 4A–D). Firstly, given the fibre-reinforced hydrogels displayed biomimetic J-shaped stress strain curves during unconfined mechanical compression (Fig. 1), we hypothesised that the mPCL microfibres used to reinforce hydrogel constructs may mimic biological tissues that contain fibrillar biopolymers which uncoil and straighten with compression [50].

Notably, overall fibre straightness was not influenced by compressive loading in any group at any compressive strain level (Fig. S3, Supplementary Data), analysed with the fibre straightness parameter as per Rezakhaniha et al. [51]. We then investigated the angular distribution of mPCL scaffold fibres by measuring fibre orientations in sCT z-stacks at discrete axial compression steps (Fig. S4, Supplementary Data). The weighted fibre angle frequency of non-deformed (0 % compressive strain) 400 μm, 800 μm and gradient construct groups show a good correspondence with the targeted 0–90° lay-down print pattern (Fig. S4C–E, Supplementary Data); however, the initial angular distribution of the 200 μm constructs appeared more disordered (Fig. S4B, Supplementary Data) as limitations surrounding MEW of small porosity scaffolds becomes increasingly difficult. As scaffolds were printed with a 0–90° lay-down print pattern, the change in weighted fibre orientation was quantified at −90° to −80°, −5°–5° and 80°–90° to determine whether compression increased global fibre alignment towards −90°, 0° or 90° in response to loading (Fig. S4F–H, Supplementary Data). Whilst some groups displayed a slight change in fibre alignment towards these orientations, these were not considered significant. Further, to determine whether there was any depth-dependent behaviour under compressive loading, we measured fibre straightness as per Rezakhaniha et al. [51] in discrete regions (top, middle and bottom of each scaffold) (Fig. 4E–H). Although fibre straightness did somewhat vary in response to loading in each zone for each construct type, the differences were, once again, not significant (Fig. 4E–H).

Next, the height of the reinforcing microfibre scaffolds did not decrease linearly with compressive strain applied (Fig. 4I). This suggested that the expansion of the hydrogel phase within the porous scaffold constructs accounted for the predominant proportion of strain; however, the scaffold diameter increased nearly linearly in all groups (Fig. 4J). No significant changes were observed in scaffold pore size in monophasic constructs in response to axial compression (Fig. 4K, solid lines). To determine how each zone of the gradient scaffold responded to axial compression, zone pore sizes across were also measured at each compression strain step (Fig. 4K, dashed blue lines), showing negligible changes.

Based on the aforementioned diameter and height measurements made, estimates of the Poisson's ratio of each scaffold group were determined based on the relative axial and lateral strain ascertained from morphological measurements (Fig. 4L). While no significant differences were observed between the groups, which also exhibited large standard deviations, with the 200, 400, 800 and gradient constructs exhibiting Poisson's ratios of 0.24 ± 0.35, 0.33 ± 0.40, 0.17 ± 0.37 and 0.24 ± 0.31, respectively, these low Poisson's ratios indicate the limited lateral expansion of the materials under compression.

### 3.5 Depth-dependent strain properties of mPCL scaffolds from sCT reconstructions

**Fig. 5:**
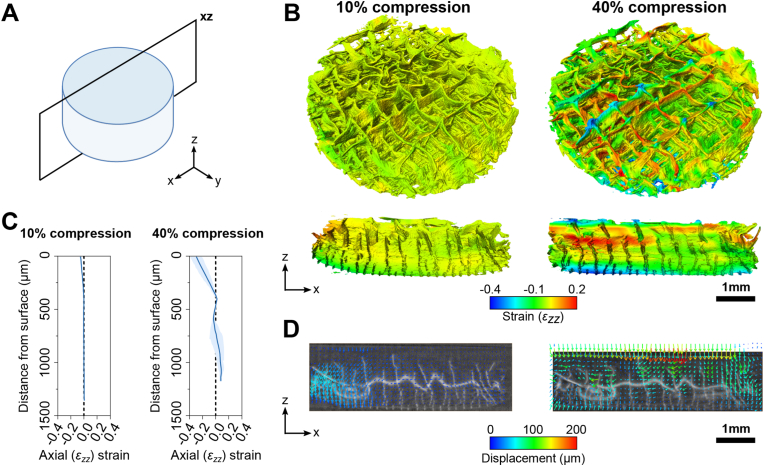
**Axial (*Ɛ*_*zz*_) strain DVC analysis of synchrotron x-ray μCT imaged gradient mPCL-reinforced hydrogel constructs at different axial compression steps.** (A) Representative diagram of scaffold showing cut-away plane location for DVC images. (B) 3D reconstructions of hydrogel constructs with *Ɛ*_*zz*_ strain superimposed on the rendered surface with cut-away views displaying magnitude of strain within the construct. (C) Plot of *Ɛ*_*zz*_ strain across the height of the reinforcing scaffold at 10 and 40 % compression steps (mean ± SD; 3 measurements, *n* = 1 construct). (D) Displacement vector fields displaying the direction and magnitude of scaffold displacement at 10 and 40 % compression steps superimposed over an orthogonal tomogram slice of the construct.

Following morphological characterisation of the 3D sCT models, 3D displacement and strain fields within the gradient constructs were calculated using DVC (Fig. 5; Fig. S5, Supplementary Data). Analysis of axial (*Ɛ*_*zz*_) strain fields (Fig. 5A) revealed that the uppermost portion of the gradient scaffold component of reinforced hydrogel constructs experiences the greatest degree of strain with 5.2 % local strain at 10 % imposed axial compression, and up to 29.8 % strain at 40 % imposed axial compression (Fig. 5B and C). Strain at the centre of the construct appeared to be negligible. However, at 40 % compression, a region of tensile strain became visible which correlates with increasingly buckled fibres at the interface between the 800 and 400 μm fibre spacing zones (Fig. 5B). DVC analysis of the construct exterior shows that axial strain is particularly concentrated at the fibre interconnection points (Fig. 5B). Further strain DVC analyses (Fig. S5, Supplementary Data) indicated that as constructs were compressed, lateral strains (*Ɛ*_*yy*_ and *Ɛ*_*xx*_) became increasingly pronounced along the fibre walls of the gradient scaffold (Fig. S5, Supplementary Data). These lateral strains were highest at the midpoint between fibre interconnection points, whilst negative lateral strain peaked at the interconnection points, indicating fibres were being stretched between them.

Displacement vectors overlaid on xz-axis tomograms show that constructs undergo downward or diagonal displacement until 40 % compression, at which point displacement appears to be in both the axial and lateral directions (Fig. 5D). The equivalent Von Mises strain shows a similar pattern as *Ɛ*_*zz*_ at 40 % compression, with strain concentrating at the upper and lower portions of the construct (Fig. S6, Supplementary Data). Correspondingly, the equivalent Von Mises strain of the gradient reinforced hydrogel construct obtained from the FE modelled gradient construct was observed to be highest at the upper portions of the construct at 40 % compression (Fig. S7, Supplementary Data). The equivalent Von Mises strain values differ between the experiment and FE model, as experimental force data for the specific experimental sample was not available and the FE model uses mean values of material parameters obtained for each fibre grid spacing. Still, the FE model highlights that separate layers exhibit similar differences with each other in both the experimental and FE modelled samples.

### 3.6 Cytocompatibility and cartilage tissue formation

**Fig. 6:**
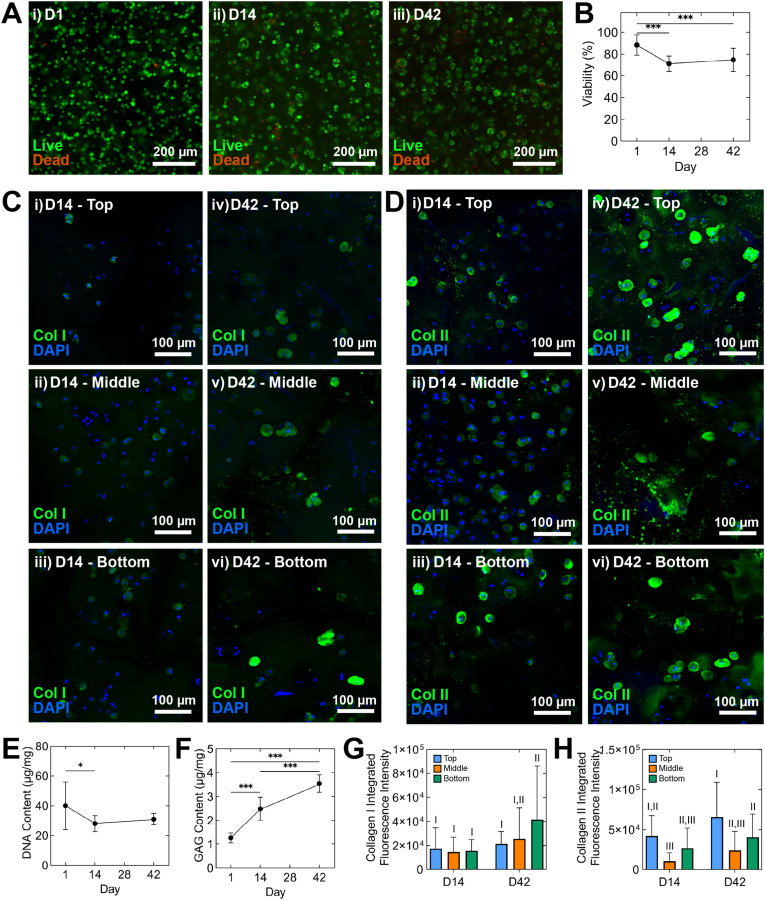
**Cytocompatibility and *in vitro* hyaline neocartilage formation in multiphasic mPCL microfibre-reinforced GelMA/HAMA constructs.** (A) Fluorescence imaging stained viability of expanded human articular chondrocytes at day 1, 14 and 42 of re-differentiation culture (living cells appear green and dead cells appear red; scale bar: 200 μm). (B) Cell viability represented as a percentage across the culture period. (C,D) Immunofluorescence images exhibiting (C) collagen I and (D) collagen II accumulation within depth-dependent zonal regions, at (i–iii) day 14 and (iv–vi) day 42 (scale bars: 100 μm). Quantification of (E) DNA and (F) GAG content at days 1, 14, and 42, respectively (3 donors, 1–2 constructs per donor) (One-way ANOVA, ∗p < 0.05, ∗∗∗p < 0.001). (G,H) Quantitative representation of integrated fluorescence intensities of (G) collagen I and (H) collagen II staining within depth-dependent regions of gradient composites; groups that do not share a Roman numeral are statistically different (two-way ANOVA, p < 0.05) (mean ± SD). (For interpretation of the references to colour in this figure legend, the reader is referred to the Web version of this article.)

Lastly, we investigated the cytocompatibility and capacity to support *in vitro* neocartilage formation of expanded human articular chondrocytes encapsulated in the gradient mPCL microfibre-reinforced GelMA/HAMA hydrogels. Aside from a slight decrease between day 1 and 14, our data indicate high cell viability throughout the 42-day culture (Fig. 6A and B). Similar to non-reinforced GelMA/HAMA constructs [43], encapsulated cells displayed rounded morphologies indicative of chondrogenic phenotypes and formation of multicellular, chondron-like structures by day 42 (Fig. 6C and D(iv–vi)). Immunofluorescence analysis demonstrated formation of cartilage-like tissue components, specifically intracellular collagen I immunoreactivity (Fig. 6C) and strong accumulation of collagen II (Fig. 6D). Using confocal immunofluorescence imaging for visualisation collagen I and II deposition within the gradient scaffold-reinforced composites, samples were stained and analysed at day 14 and 42 timepoints (Fig. 6C and D), respectively. For each sample set, microscopy images were obtained within the depth-dependent regions of the gradient composites to assess the influence of varied pore sizing on collagen I and II deposition. For collagen I (Fig. 6C–G), while a slight increase in staining was observed between the early and late timepoints, there was no statistically significant increase in integrated fluorescence intensity at day 42 compared to day 14 in the depth regions except for the bottom 200 μm region of the gradient constructs.

For collagen II (Fig. 6D–H), while increased staining at day 42 was once again observed through microscopy, quantitative analyses indicated that these differences were not significant for each depth region. Thus, despite the non-significant increase, the trend in greater collagen staining was observed, with methods to enhance this further in future work required. It must also be noted that staining for collagen II in the top and bottom regions did exhibit considerably greater measured intensity than the middle region, indicating a potential influence of nutrient diffusion. Given that no mechanical stimulation was incorporated into this study, which we intend to do in long-term culture studies in the future, we cannot in this study claim the depth-dependent behaviour of the gradient constructs under compression influenced the *in vitro* results. This will be assessed in future work as noted, to test these hypotheses further.

It must be noted in this static culture study to validate the use of the gradient composites, that the deposition of type II collagen was predominantly pericellular; however, considerable, albeit weaker, immunostaining of collagen components within the extracellular space was observed around nuclei within the composite matrix. This ECM deposition pattern indicates potentially reduced diffusion of collagen components within the densely crosslinked hydrogels, with extended culture periods and mechanical stimulation required for enhanced ECM production, as we have done in more complex *in vitro* studies previously [43]. In future work, the culture duration will be extended, while physiologically relevant mechanical stimulation, increased initial cell density, and improved incorporation of chondrogenic growth factors may also provide improved outcomes.

While a slight decrease in DNA content between day 1 and 14, corresponding with viability measures, DNA remained relatively stable (Fig. 6E) over time suggesting minimal proliferation. However, constructs supported the accumulation of GAGs (Fig. 6F) which increased significantly with culture duration, in line with our previous studies [43]. Furthermore, in future *in vitro* studies, further characterisation of the progression of mechanical properties of these constructs are required to be assessed. It is hypothesised that with increased matrix deposition within the reinforced hydrogels over long-term culture, construct stiffness will also increase, which may be further elucidated with inclusion of *in situ* mechanical stimulation during culture.

Previously, we have investigated the *in vitro* performance of GelMA/HAMA hydrogels as scaffolds for chondrocyte culture, both with and without scaffold reinforcement [11]. These studies as referenced, demonstrate increased deposition of collagen components within the ECM of the monophasic scaffold reinforced hydrogel constructs, in comparison to the unreinforced hydrogel control groups, hypothesised to be associated with the increased stiffness. The results presented in the current study correspond well with the monophasic scaffold reinforced data, with future studies required to investigate the influence of mechanobiological stimulation through the use of our mechanical stimulation bioreactor [43]. Based on the culmination of data obtained across the current study and our previously published results, it is hypothesised that, the depth-dependent compression of the gradient scaffold-reinforced constructs presented herein, in combination with bioreactor culture, will lead to enhanced cartilage-like tissue generation *in vitro*. Such work is essential to take this work further, and is planned for future experiments.

## 4 Discussion

Photocrosslinkable hydrogels have been extensively investigated for tissue engineering applications due to their modifiable mechanical and chemical properties. Whilst previously shown to support chondrocyte redifferentiation and ECM synthesis for articular cartilage repair applications, unmodified hydrogels do not possess adequate mechanical strength to support joint load bearing *in vivo*. To overcome this, we have previously shown that MEW fibre-reinforced hydrogels exhibit significantly enhanced construct stiffness, often nearing the bulk mechanical properties of native articular cartilage [[12], [13], [14], [15],^52^,^53^]. However, it is unclear whether depth-dependent strain effects in hydrogels reinforced with monophasic or gradient MEW mPCL scaffolds have a beneficial impact on compression response. Furthermore, whilst it is hypothesised that fibre behaviour under compressive strain influences mechanical response, this has not been demonstrated. The aim of this study therefore was to observe and quantitatively analyse these mechanisms through various techniques to gain a greater understanding of how fibre-reinforcement affects the compression properties of hydrogels toward improved bioengineered cartilage repair strategies.

In this study, we fabricated reinforced hydrogels with monophasic mPCL scaffold with either 200, 400 and 800 μm fibre spacing throughout construct depth, or using a hierarchically structured scaffold with fibre spacing organised in a descending gradient from the construct surface (from 800, 400 to 200 μm fibre spacing) (Fig. 1, Fig. 2). Fibre diameter and spacing were well-controlled, with limited variability between scaffold types of different fibre spacings. However, monophasic scaffolds printed with a targeted 200 μm fibre spacing exhibited significant variance in fibre diameter and fibre spacing compared with other groups. This is a possible structural explanation leading to the observed greater variance in the fibril network moduli of the 200 μm group reflected in FE simulations (Fig. 3).

The reduced accuracy in construct conformity in the 200 μm scaffold group may be attributed to the fact that charge is dissipated by the deposition of fibres on the collector; however, residual charge often remains within the scaffold creating an electret effect, hence reducing the accuracy of printed fibres due to electrostatic repulsion [31]. Strategies to mitigate disorganised fibre deposition include using a highly conductive collector to reduce interfibre distance limitations [54]. Additionally, incremental increases in print head height and applied voltage have been investigated to overcome the diminishing electrical field during thicker scaffold production [55]. Accordingly, the development of MEW devices with greater control are part of our future work to facilitate the accurate and reproducible manufacture of small-porosity scaffolds with a height of >1 mm.

One line of research is directed towards, ‘functional tissue engineering’, which aims to identify the critical biomechanical elements of the native tissue and incorporate them into the design and fabrication of tissue-engineered constructs [56]. We analysed the compression properties of fibre-reinforced hydrogels within this context to determine their suitability for articular cartilage repair applications. Compression of MEW fibre scaffolds alone (without hydrogel) causes buckling and collapse of the overall fibre structure [12,15], whilst unreinforced GelMA and GelMA/HAMA-based hydrogels possess limited compressive moduli (typically <100 kPa) [34,57]. However, in concert, these two materials synergistically enhance the overall compressive strength of the construct [13]. Here, incorporation of mPCL scaffolds increased hydrogel stiffness by up to 22-fold over hydrogels alone, with the increase inversely correlated with the mPCL scaffold pore size. Although the absolute equilibrium modulus of the composite hydrogels remained lower than that of native articular cartilage, the gradient structure effectively reproduced the depth-dependent trend in stiffness and strain response, representing an important step toward biomimetic mechanical properties of these constructs.

Multiple studies have observed a similar correlation between fibre spacing and compressive strength in fibre-reinforced hydrogels [[12], [13], [14]]. It is hypothesised that the axial compressive force is transferred to the transverse fibres and smaller pore sizes afford a greater reinforcing filler ratio [12]. DIC (Fig. 2) and DVC (Fig. 4, Fig. 5; Figs. S5 and 6, Supplementary Data) analyses supported this hypothesis, with the hydrogel matrix experiencing a large degree of lateral and shear strain under axial loading, pushing against the fibre walls, whilst the mPCL fibres exhibit tensile strain along their length. Thus, hydrogel expansion is counteracted and contained within the mPCL fibres, leading to fluid pressurisation similar to that in the crosslinked collagen network in cartilage. Notably, the data demonstrates that axial compression does not result in significant increases in scaffold pore size or fibres straightness. Additionally, the FE simulations (Fig. 3) verified the fluid pressurisation due to the fibre network, demonstrating significant interstitial fluid pressurisation in the reinforced composites under loading, while the fluid pressurisation was minimal in hydrogel only. Thus, our data suggest that the mechanical mismatch between the comparatively stiff mPCL scaffold and soft hydrogel phase may be required to achieve this level of mechanical enhancement. This was also confirmed by parametric FE analysis (Fig. S8, Supplementary Data), indicating that the stiffness difference between hydrogel and fibre scaffold is essential for both initial pressurisation and fast relaxation.

Soft tissues that contain biopolymers such as collagen typically exhibit a J-shaped stress-strain response to loading as fibres undergo progressive uncoiling and unkinking [58]. All reinforced hydrogel constructs in the current study exhibited this J-shaped stress-strain curves in response to axial compression. This response may be attributed to the distribution of strain between the hydrogel within the scaffold pores, counteracting excessive lateral expansion. This could be improved by replicating Bas et al. [32] who made reinforced hydrogel constructs with wavy MEW mPCL scaffolds printed to mimic soft tissue mechanics. Wavy, biomimetic fibre structures resisted tensile strains of up to 46 %.

To determine how compression affected fibre behaviour in our constructs, we measured both the global fibre angle and fibre straightness (Figs. S3 and S4, Supplementary Data) from sCT tomograms and 3D reconstructions, respectively. However, no significant changes were observed across groups at various compressive strains, given the high initial conformity to the intended crosshatch scaffold design. Further, local fibre straightness did not appear to change significantly in response to loading across the depth of the hydrogel constructs, with this most likely due to the orthogonal design pattern of the constructs used herein, whereas wavy architecture may induce significant changes in fibre orientation and straightness [25].

Native articular cartilage can be compressed 15–45 % in response to physiological long-term or static loading [59]. Single-network hydrogels are typically brittle with low-toughness because they do not possess effective stress dissipation mechanisms [60]. Whilst fibre-reinforcement of hydrogels significantly enhances stiffness, they exhibit brittleness under high compressive loading [13]. Here, the fibre-reinforced GelMA/HAMA hydrogel constructs resisted failure to ∼22 % (200 μm group) and ∼42 % (800 μm group) compressive strain (Fig. 1); however, failure stress was two orders of magnitude lower than that of the native tissue. Cyclic testing may reveal additional data pertinent to the long-term stability and mechanical performance of our constructs. Limitations in the experiment design restricted such testing to be performed in this study, though it will be incorporated in future work, as appropriate.

In a previous study [13], compression beyond 35 % strain in GelMA hydrogels reinforced with 200 μm-spaced fibres greatly reduced the resistance to further compressive strain. It was hypothesised that either hydrogel fracture or delamination of the fibre junctions was responsible [13]. High-resolution sCT imaging of the reinforced hydrogel constructs in our study has revealed that fibre walls indeed buckle or kink at specific locations, such as fibre regions with pre-existing defects or misalignments (Fig. 4). For hydrogels reinforced with gradient mPCL scaffolds, this weak point appears to be the interface between the 400 and 800 μm fibres spacing region. Furthermore, the fibre wall does not perfectly trap the hydrogel matrix within the scaffold. During the MEW printing process horizontal pores are created by the polymer jet dragging over raised fibre interconnection points. The large lateral and shear strains (Fig. S5, Supplementary Data) generated within the construct under compression may force the hydrogel through these horizontal pores, increasing interfacial shear which may cause localized delamination of the hydrogel matrix.

For functional articular cartilage tissue engineering, the relaxation rate of viscoelastic biomaterials is an important design parameter. Lee et al. [46] have previously shown that chondrocytes cultured in fast relaxing, low molecular weight alginate hydrogels synthesised interconnected volumes of cartilage matrix. While in our tests, a relaxation time of 600 s was not sufficient for the constructs to reach full stress equilibrium, all mPCL scaffold-reinforced hydrogel groups exhibited a significantly faster short-term stress-relaxation response (Fig. 2) than unreinforced hydrogels. This is likely to due to the rapid pressurisation of the confined hydrogel phase which increases the rate of fluid exudation, as evidenced by the greater degree of longer-term relaxation compared to the hydrogel only group [53]. It must be noted that, while the major viscoelastic differences between groups were captured here, longer experimental relaxation times (>10 min) may reveal additional slow relaxation behaviour, to be performed in future work where appropriate. Moreover, a key advantage of the inverse FE analysis and fibril-reinforced material model is that it can capture the equilibrium response even when the full experimental relaxation is not achieved (Table S2, Supplementary Data).

In the reinforced hydrogel construct models, the stress-relaxation response is strongly driven by pressurised fluid. This results in much higher force responses, compared to the hydrogel-only constructs (Fig. 3). Thus, in the computational FRPE models, the viscoelastic behaviour could be considered with fluid flow-dependent viscoelasticity, and the non-fibrillar matrix was considered as hyperelastic, allowing for reduction of the number of optimised FRPE material parameters. In the unreinforced hydrogel constructs, fluid pressurisation remained lower, and the viscoelastic stress-relaxation behaviour had to be defined as fluid flow-independent as poroelastic models were not successful in capturing the experimentally measured relaxation ($R^{2}=0.90\;\pm 0.04\text{,}$ data not shown). Thus, the solid unreinforced matrix was modelled as a hyperviscoelastic material for the unreinforced hydrogel constructs.

While the simplified fibre network geometry in the current FE model does not permit analysis of highly localised mechanical effects of individual fibres in the fibre scaffold, it enables direct comparison of the obtained material properties with previously reported values for native articular cartilage on the macroscopic level. The FRPE material stiffness parameters determined for reinforced hydrogel constructs were lower than values reported for articular cartilage [[27], [28], [29], [30]], and the initial permeability of reinforced constructs were higher than healthy human tibial and femoral cartilage [28,29]. This demonstrates that key differences between native and fibre-reinforced constructs remain in the current model. However, the material parameters are within the range of values previously reported for osteoarthritic human articular cartilage [[28], [29], [30]], demonstrating compatibility with the osteoarthritic tissue environment. Moreover, in the presence of chondrocytes, fibre-reinforced composites quickly accumulate significant amounts of mechanically functional hyaline extracellular matrix, and over time, remodel to become increasingly similar to native tissue [11], suggesting that these parameters are likely to progressively assimilate to native tissue over time. The fibril stiffness parameters (initial and strain-dependent fibril network modulus) are measures of the construct's ability to resist rapid deformation, which affect the peak forces during stress-relaxation [[27], [28], [29], [30]]. The strain-dependent fibril modulus was significantly higher in the 400 μm group than in the 800 μm group, likely due to increased fibre waviness (Fig. 1). This is further supported by the higher IFLS in the 400 μm group, despite similar permeability (Fig. 3). Stiffer fibres resist tension more effectively, altering relaxation behaviour. At equilibrium, once fluid flow ceases, stiffness is governed by the non-fibrillar matrix modulus [26].

The material parameters of the unreinforced hydrogel constructs were obtained separately for each step to characterise the strain-dependent stress-relaxation behaviour. The Prony series dimensionless shear modulus (ratio between instantaneous and equilibrium shear modulus) was significantly higher and increased with compressive strain applied (Fig. 3). In our model, the bulk modulus was set equal to the shear modulus, and therefore the observed difference similarly applies to the ratio between the instantaneous and equilibrium bulk modulus. Correspondingly, the Prony series characteristic time, which describes the rate of relaxation, increased significantly with increasing compression across all the compared steps. Taken together, these results suggest that the viscosity and energy dissipation of unreinforced hydrogels correlate with the applied strain magnitude.

It is well established that the heterogenous architecture of native articular cartilage ECM creates depth-dependent anisotropies and inhomogeneities in the biomechanics of the tissue [61]. *In vitro* mechanical assessments of articular cartilage explants show that axial strain is primarily localised near the articular surface and progressively decreases towards the deep zone in the native tissue which reflects the increasing compressive modulus moving away from the articulating surface [[62], [63], [64]]. Strain analysis of our mPCL-reinforced hydrogels show that both the hydrogel and fibre phase of the gradient scaffold composites closely mimics this strain behaviour observed in DIC analyses (Fig. 2). A limitation of the DIC analyses in this study was that we were unable to directly validate these findings against native tissue analyses due to experimental constraints. In future studies, it may be required to perform comparative DIC imaging and analyses on native cartilage tissue samples for additional validation. In line with DIC methods and results for analysis of cartilage tissue previously published by us [65,66], these analyses will be incorporated into planned mechanostimulation bioreactor culture studies utilising the gradient scaffold reinforced constructs, given the hypothesis is that the compression properties will be further enhanced through dynamic *in vitro* chondrocyte studies [43]. Similar depth-dependent strain behaviour was also observed in the FE modelled gradient construct (Fig. S6, Supplementary Data), consistent with the increasing compressive modulus of the mPCL gradient scaffold [14]. However, articular cartilage may show greater variability of axial strains *in vivo* due to differences in joint morphology and flexion angle as well as the influence of the surrounding soft tissue [67,68].

In articular cartilage, collagen fibres in the superficial zone are organised parallel to the articular surface to provide a high degree of tensile strength. However, the superficial zone also exhibits the greatest lateral strain in response to compression despite the tangential tensile modulus decreasing as collagen fibres become increasingly perpendicularly aligned to the articular surface with tissue depth [62,63,69]. Hydrogels in all construct groups exhibited a degree of lateral strain (*Ɛ*_*xx*_ or *Ɛ*_*yy*_) (Fig. S5, Supplementary Data), which is likely due to the lateral expansion of the hydrogel in response to loading and being imaged prior to relaxation [67]. Lateral strain was nearly constant through the depth of the monophasic constructs, likely due to the scaffold's consistent tensile modulus. In the gradient constructs, lateral strain was concentrated in the 800 and 400 μm fibre spacing zones, with limited lateral strain observed in the 200 μm fibre zone. This indicates that the gradient scaffold composites possessed depth-dependent compression behaviour in biomimetic patterns similar to that of cartilage, with further developments required to fully recapitulate native tissue mechanics in these models. Shear strain distributions also exhibit depth-dependent profiles in articular cartilage, with the large differences in mechanical properties at the cartilage-bone interface, concentrating shear strain in the deep zone [63].

The gradient scaffold group displays similar properties; while the monophasic scaffolds display no such depth-dependent features due to their consistent fibre spacing. Together these physical and computational results validate the mechanical relevance of the gradient scaffold design for future application in cartilage repair, with positive indications for chondrogenic cell culture *in vitro* further supporting these results (Fig. 6). The GelMA/HAMA hydrogels reinforced with gradient mPCL scaffolds successfully supported high cell viability throughout the 42-day culture period, with positive chondrogenic cell growth evidenced by increases in DNA content. Hyaline-like cartilage ECM components were observed to form across the culture period, where the deposition of collagen I and II after 42 days was substantial. Thus, while further studies are required to assess additional factors of neocartilage formation using the constructs produced herein, the capability of these constructs to promote functional tissue regeneration *in vitro* has been demonstrated.

Furthermore, this study presents a significant advancement in the field through the novel methodological approach to the characterisation of reinforced hydrogel scaffolds. Using synchrotron x-ray μCT (sCT) imaging under compressive loading, combined with DIC and DVC, as well as comparative analyses performed with the fibril-reinforced poroelastic FE modelling, we demonstrate a unique framework for analysing strain distribution in soft biomaterials. To our knowledge, this application of sCT imaging and modelling has not been previously reported, offering new insights into scaffold mechanics relevant to cartilage regeneration.

## 5 Conclusion

This study shows that sCT, DVC, mechanical analyses, DIC, and FE simulations offer detailed insights into fibre-reinforced hydrogels' strain behaviour *in vitro*. GelMA/HAMA hydrogels with gradient mPCL scaffolds replicate native cartilage's depth-dependent strain patterns, creating advanced scaffolds for tissue engineering, supporting chondrogenic growth and ECM *in vitro*. Gradient scaffolds enhance stress-relaxation, key for maximising matrix synthesis in cartilage constructs. While fibre straightening was limited, sCT and DVC effectively characterised fibre behaviour, supporting future computational FE analyses. These simulations underline the role of reinforcing fibres role in promoting physiological fluid pressurisation under compression, offering insights into mechanical behaviour. Future preclinical studies will be essential to assess these constructs' capacity for functional tissue regeneration *in vivo*, taking these models beyond *in vitro* model applications, toward the ultimate aim of developing improved MACI treatments.

## CRediT authorship contribution statement

**Stephen Pahoff:** Writing – original draft, Visualization, Validation, Methodology, Investigation, Formal analysis, Data curation, Conceptualization. **Angus Weekes:** Writing – review & editing, Writing – original draft, Visualization, Formal analysis, Data curation. **Heta Mertano:** Writing – review & editing, Writing – original draft, Visualization, Methodology, Investigation, Formal analysis, Data curation, Conceptualization. **Johannes J. Braig:** Writing – review & editing, Methodology, Investigation, Formal analysis. **Michael W.M. Jones:** Writing – review & editing, Methodology, Funding acquisition, Conceptualization. **Anton Maksimenko:** Writing – review & editing, Resources, Methodology, Investigation. **Janne T.A. Mäkelä:** Writing – review & editing, Supervision, Methodology, Formal analysis, Data curation, Conceptualization. **Petri Tanska:** Writing – review & editing, Methodology, Formal analysis. **Rami K. Korhonen:** Writing – review & editing, Supervision, Software, Methodology, Funding acquisition, Conceptualization. **Juha Töyräs:** Writing – review & editing, Supervision, Methodology, Funding acquisition, Conceptualization. **Dietmar W. Hutmacher:** Writing – review & editing, Supervision, Resources, Project administration, Funding acquisition, Conceptualization. **Travis J. Klein:** Writing – review & editing, Supervision, Resources, Project administration, Methodology, Funding acquisition, Formal analysis, Data curation, Conceptualization. **Christoph Meinert:** Writing – review & editing, Supervision, Resources, Project administration, Methodology, Investigation, Funding acquisition, Formal analysis, Data curation, Conceptualization.

## Ethics statement

The experimental procedures described herein were performed in accordance with Queensland University of Technology (QUT), University Human Research Ethics Committee (UHREC) approval. Articular cartilage explants were obtained with informed consent and institutional ethical clearance from three separate donors all undergoing total knee arthroplasty surgery for osteoarthritis (QUT UHREC #EC00171 and the Prince Charles Hospital Human Research Ethics Committee #EC00168).

## Declaration of competing interest

The authors declare the following financial interests/personal relationships which may be considered as potential competing interests: Travis Klein reports a relationship with Gelomics that includes: board membership and equity or stocks. Christoph Meinert reports a relationship with Gelomics that includes: board membership, employment, and equity or stocks. Dietmar Hutmacher reports a relationship with Gelomics that includes: board membership and equity or stocks. If there are other authors, they declare that they have no known competing financial interests or personal relationships that could have appeared to influence the work reported in this paper.

## Data Availability

Data will be made available on request.
