# Supplementary material for: Engineering biomimetic zonal properties in fibre-reinforced hydrogels for functional cartilage tissue engineering in vitro
Source: Mater Today Bio. 2025 Nov 11;35:102550. doi: 10.1016/j.mtbio.2025.102550 (PMC12663666; doi:10.1016/j.mtbio.2025.102550)
Supplement: Multimedia component 1 [file mmc1.docx]

**SUPPLEMENTARY DATA**

Engineering biomimetic zonal properties in fibre-reinforced hydrogels for functional cartilage tissue engineering *in vitro*

*Stephen Pahoff, Angus Weekes, Heta Mertano, Johannes J. Braig, Michael W. M. Jones, Anton Maksimenko, Janne T. A. Mäkelä, Petri Tanska, Rami K. Korhonen, Juha Töyräs, Dietmar W. Hutmacher, Travis J. Klein, Christoph Meinert*

Queensland University of Technology (QUT), Brisbane, QLD, Australia

## Supplementary Methods

**Material model of reinforced hydrogel constructs**

A fibril-reinforced poroelastic (FRPE) material consisting of a fibrillar matrix and a poroelastic non-fibrillar matrix filled with fluid constituted the reinforced hydrogel material [1]. The total stress was defined as the sum of fibrillar and non-fibrillar stress as follows:

| $\boldsymbol{\sigma}_{\mathrm{tot}}=\boldsymbol{\sigma}_{f}+\boldsymbol{\sigma}_{\mathrm{nf}}-p\mathbf{I}$, | (1) |
| --- | --- |

where $\boldsymbol{\sigma}_{f}$ is the fibrillar stress tensor, $\boldsymbol{\sigma}_{\mathrm{nf}}$ is the non-fibrillar stress tensor, $p$ is the fluid pressure and $\mathbf{I}$ is the unit tensor. Darcy’s law was used to describe the fluid flow in the porous solid matrix as

| $q = -k\nabla p,$ | (2) |
| --- | --- |

where $q$ is the fluid flow flux, $k$ is the permeability of the material and $\nabla p$ is the fluid pressure gradient. The permeability, $k$, depends on the void ratio $e$ as

| $k=k_{0}\left( \frac{1+e}{1+e_{0}} \right)^{M},$ | (3) |
| --- | --- |

where $k_{0}$ and $e_{0}$ are the initial permeability and void ratio, respectively, and $M$ is the strain-dependent permeability factor [2]. The non-fibrillar matrix was modelled as a compressible Neo-Hookean material, for which the stress is expressed as

| $\boldsymbol{\sigma}_{\text{nf}}=\frac{1}{2}K_{\mathrm{nf}}\left( J-\frac{1}{J} \right)\text{I}+\frac{G_{\mathrm{nf}}}{J}\left( \text{F}\text{F}^{\text{T}}-J^{2/3}\text{I} \right),$ | (4) |
| --- | --- |

where $\text{F}$ is the deformation gradient tensor, $J$ is the determinant of $\text{F}$, and $K_{\mathrm{nf}}$ and $G_{\mathrm{nf}}$ are the bulk and shear moduli of the non-fibrillar matrix, respectively [3]. The bulk and shear moduli are expressed as

| $K_{\mathrm{nf}}=\frac{E_{\text{nf}}}{3\left( 1-2\nu_{\text{nf}} \right)},$ | (5) |
| --- | --- |
| $G_{\mathrm{nf}}=\frac{E_{\text{nf}}}{2\left( 1+\nu_{\text{nf}} \right)},$ | (6) |

where $E_{\text{nf}}$ is the non-fibrillar matrix modulus and $\nu_{\text{nf}}$ is Poisson’s ratio. The fibril network consisted of 4 primary and 13 secondary fibrils [1,4], and the total fibrillar stress tensor is defined as the sum of primary and secondary fibril stress tensors

| $\boldsymbol{\sigma}_{f,p}= C\sum_{i=1}^{4} \sigma_{f,i}\vec{e}_{f,i}\bigotimes\vec{e}_{f,i}$ | (7) |
| --- | --- |
| $\boldsymbol{\sigma}_{f,s}= \sum_{i=5}^{17} \sigma_{f,i}\vec{e}_{f,i}\bigotimes\vec{e}_{f,i}$ | (8) |
| $\boldsymbol{\sigma}_{f}= {\boldsymbol{\sigma}_{f,p}\boldsymbol{+}\boldsymbol{\sigma}}_{f,s}$ | (9) |

where $\boldsymbol{\sigma}_{f,p}$and $\boldsymbol{\sigma}_{f,s}$ are the primary and secondary fibrillar stress tensors, respectively, $C$ is the ratio of primary and secondary fibrils, $\sigma_{f,i}$ is the stress in *i*:th fibril and $\vec{e}_{f,i}$ is the orientation vector of that fibril. The ratio of primary and secondary fibrils was set to constant, $C=12.16$ [1,5]. The stress in individual fibril in tension was defined as

| $\sigma_{\text{f}}\boldsymbol{=}\frac{1}{2}E_{\text{f}}^{\varepsilon}\varepsilon_{\text{f}}^{2}+E_{\text{f}}^{0}\varepsilon_{\text{f}},$ | (10) |
| --- | --- |

where $\varepsilon_{\text{f}}$ is the fibril strain, $E_{\text{f}}^{0}$ is the initial fibril network modulus and $E_{\text{f}}^{\varepsilon}$ is the strain-dependent fibril network modulus. The fibrillar stress was set to zero for compression [6].

**Material model of unreinforced hydrogel constructs**

The unreinforced hydrogel constructs were modelled as a poroviscoelastic material, consisting of a fluid and porous hyperviscoelastic solid matrix [7,8]. The total stress was defined as

| $\boldsymbol{\sigma}_{\mathrm{tot}}=\boldsymbol{\sigma}_{s}-p\mathbf{I}$, | (11) |
| --- | --- |

where $\boldsymbol{\sigma}_{s}$ is the solid stress tensor, $p$ is the fluid pressure and $\mathbf{I}$ is the unit tensor. Fluid flow and permeability *k* were defined the same as for the fibril-reinforced material (Eqs. 2 and 3, respectively). Correspondingly, the solid matrix was modelled as compressible neo-Hookean material, for which the stress is defined as

| $\boldsymbol{\sigma}_{\text{s}}=\frac{1}{2}K_{R}(t)\left( J-\frac{1}{J} \right)\text{I}+\frac{G_{R}(t)}{J}\left( \text{F}\text{F}^{\text{T}}-J^{2/3}\text{I} \right),$ | (12) |
| --- | --- |

where $\text{F}$ is the deformation gradient tensor, $J$ is the determinant of $\text{F}$ and $K_{R}(t)$ and $G_{R}(t)$ are the bulk and shear relaxation moduli at time *t*, respectively. The fluid-flow independent viscoelasticity of the material was described with a one-term Prony series:

| $g_{R}\left( t \right)=1-\bar{g}_{1}\left( 1-e^{-t/\tau_{1}} \right),$ | (13) |
| --- | --- |
| $k_{R}\left( t \right)=1-\bar{k}_{1}\left( 1-e^{-t/\tau_{1}} \right),$ | (14) |

where $g_{R}\left( t \right)$ and $k_{R}\left( t \right)$ are the normalised shear and bulk relaxation moduli ($g_{R}\left( t \right)=G_{R}(t)/G_{0}$and $k_{R}\left( t \right)=K_{R}(t)/K_{0}$ where $G_{0}$ and $K_{0}$ are instantaneous shear and bulk moduli, respectively), $\bar{g}_{1}$ and $\bar{k}_{1}$ are the Prony constants corresponding to dimensionless shear and bulk relaxation moduli and $\tau_{1}$ is the viscoelastic relaxation time. For practical reasons of Abaqus, Prony series was implemented using equilibrium shear and bulk relaxation moduli $G_{\infty}$and $K_{\infty}$ ($G_{\infty}=G_{0}\left( 1- \bar{g}_{1} \right)$ and $K_{\infty}=K_{0}\left( 1- \bar{k}_{1} \right)$, respectively). The shear and bulk relaxation moduli are expressed as

| $G_{\infty}=\frac{E_{\text{s}}}{2\left( 1+\nu_{\text{s}} \right)},$ | (15) |
| --- | --- |
| $K_{\infty}=\frac{E_{s}}{3\left( 1-2\nu_{\text{s}} \right)},$ | (16) |

where $E_{\text{s}}$ is the solid matrix modulus and $\nu_{\text{s}}$ is Poisson’s ratio.

**Finite Element Analysis**

Optimisation of the material parameters was carried out by fitting the simulated axial loading to the experimentally measured loading data. The optimised material parameters for the mPCL-reinforced hydrogel constructs were initial fibril network modulus, strain-dependent fibril network modulus, non-fibrillar matrix modulus, initial permeability and strain-dependent permeability factor ($E_{\text{f}}^{0}, E_{\text{f}}^{\varepsilon}, E_{\text{nf}}, k_{0}$ and $M$, respectively)*.* For unreinforced hydrogel constructs, the optimised parameters were solid matrix modulus, initial permeability, Prony constant corresponding to dimensionless shear modulus and Prony series characteristic relaxation time ($E_{\text{s}}, k_{0}, \bar{g}_{1}$ and $\tau_{1}$, respectively). The first step of the three-step test was regarded as a pre-step to ensure the contact between the sample and the device. For the mPCL-reinforced hydrogel constructs, the simulation was fitted to the second and third relaxation step simultaneously, whereas, for the plain hydrogel constructs, it was fitted separately to steps from two to four. This approach was used as the constructs seemed to possess a strain-dependent, nonlinear peak stress-relaxation behaviour: by optimising the material parameters for separate stress-relaxation steps, we could find out which parameters control the nonlinear behaviour. The optimisation was conducted in MATLAB (R2020a, The MathWorks Inc., Natick, MA, USA) with Nelder-Mead Simplex algorithm (*fminsearch* function)[9]. The objective function was defined as the sum of mean square error and the absolute difference between the peak and equilibrium force values of the optimised steps as follows:

| $\delta F= \frac{1}{n} \sum_{i=1}^{n} \left( \frac{F_{i}^{\mathrm{model}}-F_{i}^{\text{exp}}}{F_{i}^{\text{exp}}} \right)^{2}+\sum_{j} \left\vert\max F_{j,p}^{\text{model}}-\max F_{j,p}^{\text{exp}} \right\vert+\sum_{j} \left\vert\max F_{j,eq}^{\text{model}}-\max F_{j,eq}^{\text{exp}} \right\vert$ | (17) |
| --- | --- |

where $F_{i}^{\mathrm{model}}$ and $F_{i}^{\exp}$ are simulated and measured force values, $F_{j,p}^{\text{model}}$ and $F_{j,p}^{\text{exp}}$ are the peak force values for stress-relaxation steps, $F_{j,eq}^{\text{model}}$ and $F_{j,eq}^{\text{exp}}$ are the corresponding equilibrium force values for the steps, $n$ is the total number of data points, and *j* are the optimised steps (for reinforced hydrogel constructs, $j=2, 3$, and for plain hydrogel constructs, $j=2,$ $j=3$or $j=4$).

Poisson’s ratio of the non-fibrillar matrix for the FRPE model and solid matrix of the unreinforced hydrogels was set to constant, 0.35, for all samples, based on experimentally measured values of GelMA-HAMA hydrogels[10]. The initial fluid fraction was set to 0.78, corresponding to the initial void ratio of 3.54. Additionally, Prony constants of the normalised shear and bulk relaxation were set equal. The strain-dependent permeability factor for unreinforced hydrogel constructs was set to constant, $M=1$, based on preliminary optimisations, because the material parameters were optimised one step at a time.

## Supplementary Figures

**Synchrotron (sCT) setup**


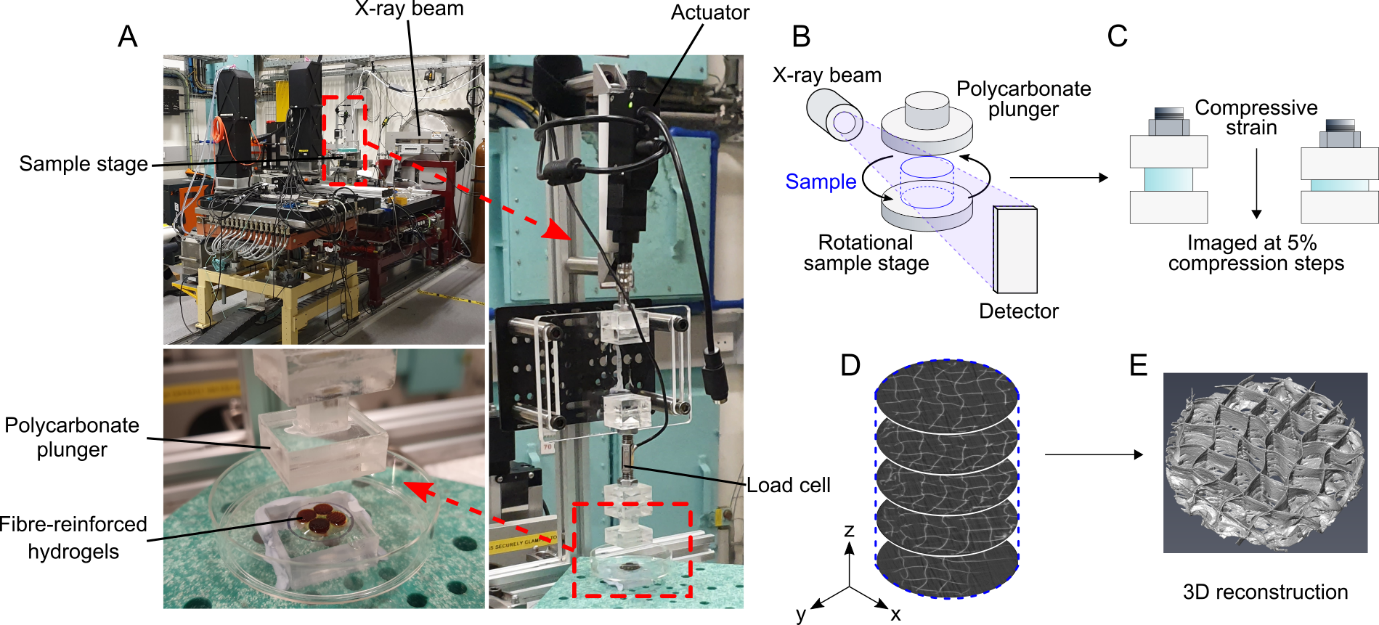


**Figure S1. Synchrotron x-ray µCT setup.** (A) The Imaging and Medical Beamline (IMBL) hutch setup for synchrotron x-ray µCT imaging of compressed mPCL fibre-reinforced GelMA-HAMA hydrogels. (B) Schematic of imaging setup showing how reinforced hydrogel samples were imaged on a 360° rotational stage (C) at each 5% compressive strain step. (D) Images were reconstructed, segmented, and processed before (E) 3D reconstruction and subsequent analysis.

**DIC image analysis of reinforced hydrogel constructs in all strain directions**


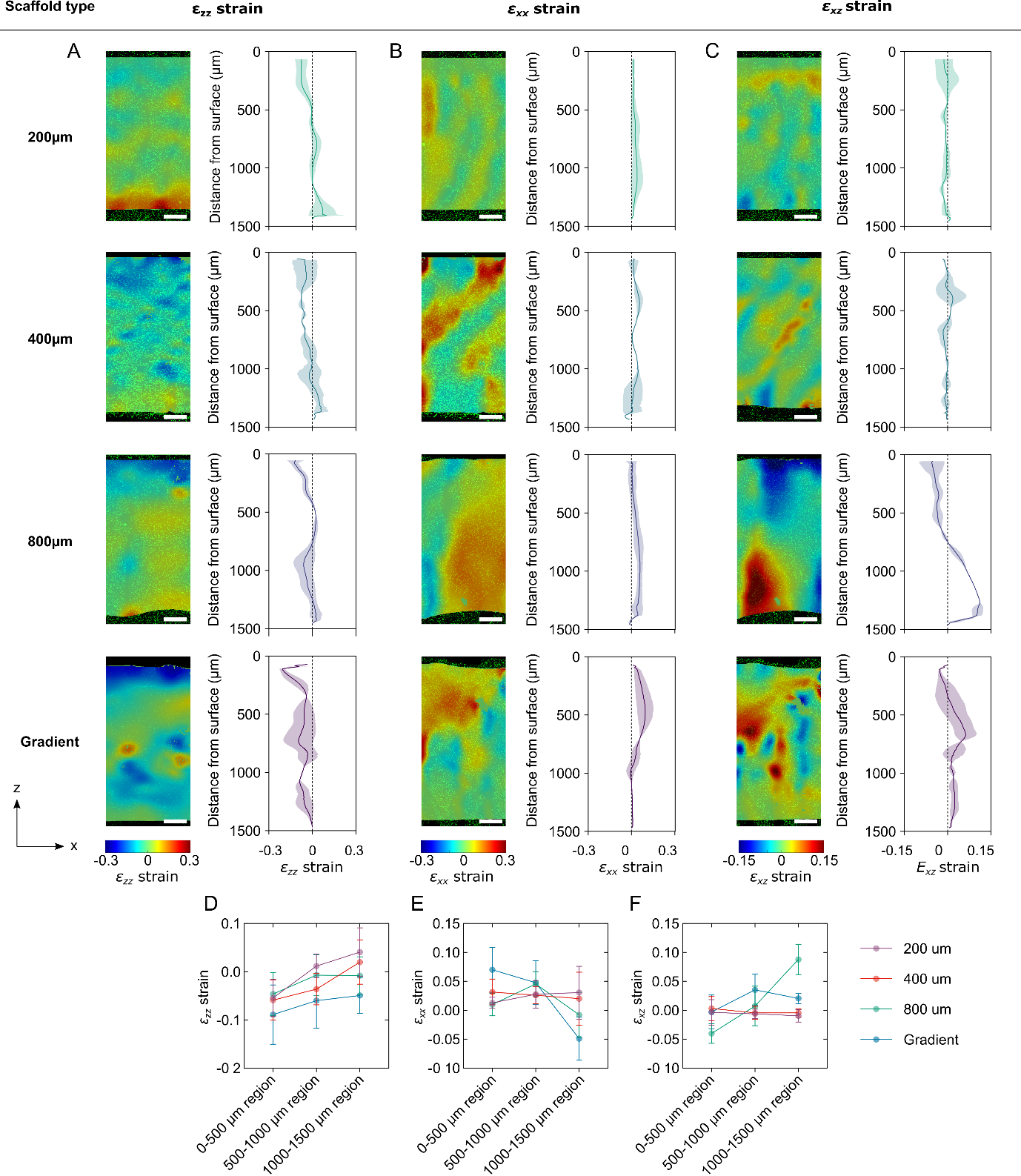


**Figure S2. Digital image correlation strain analysis of the hydrogel phase in mPCL microfibre-reinforced hydrogel constructs under axial compression.** Depth-dependent strains were measured at peak compressive strain (20%). (A-C) Representative images of compressed mPCL-reinforced GelMA/HAMA hydrogels with superimposed strain visualisation. Line graphs represent strain across the depth of the construct (mean = line, standard deviation = lighter shading; *n* = 2 per construct type; scalebar = 200 µm). (D-F) Strain profiles were divided into 500 µm regions (0-500, 500-1000 and 1000-1500 µm from construct surface, respectively) to quantify and compare the depth-dependent changes in strain between scaffolds groups (mean ± SD, *n* = 2 per construct type).

**Fibre straightness assessment from sCT reconstructions**


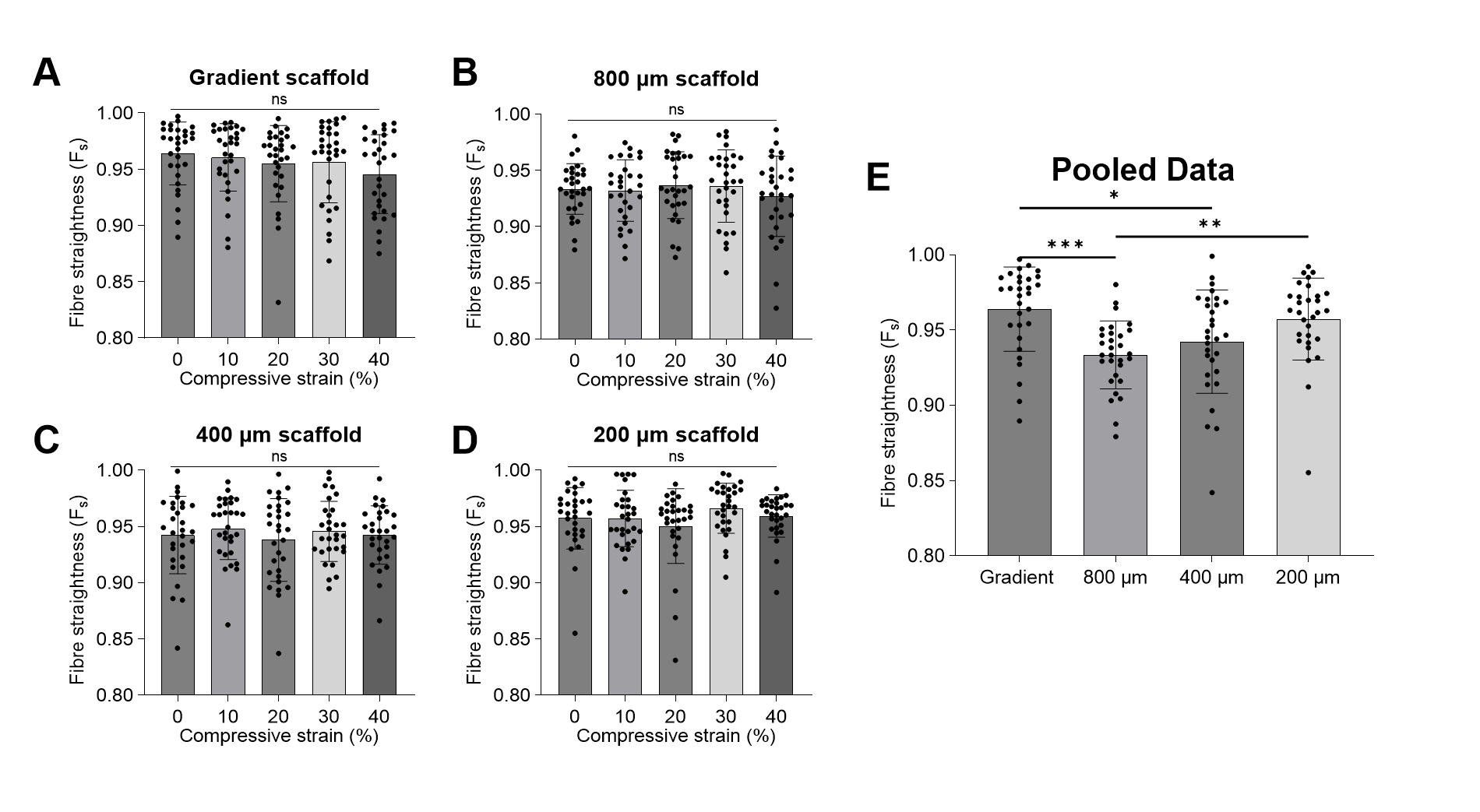


**Figure S3. Fibre straightness of mPCL fibres within scaffold-reinforced GelMA-HAMA hydrogels under axial compression.** (A-D) Quantified fibre straightness parameter (F_s_) under different compressive strains for the entire scaffold (mean ± SD; n = 2 per construct type, n = 15 fibres per scaffold; one-way ANOVA). (E) Pooled fibre straightness parameter (F_s_) for each geometry at 0% compressive strain (mean ± SD; n = 2 per construct type, n = 15 fibres per scaffold; one-way ANOVA).

**Fibre angle assessment from sCT reconstructions**


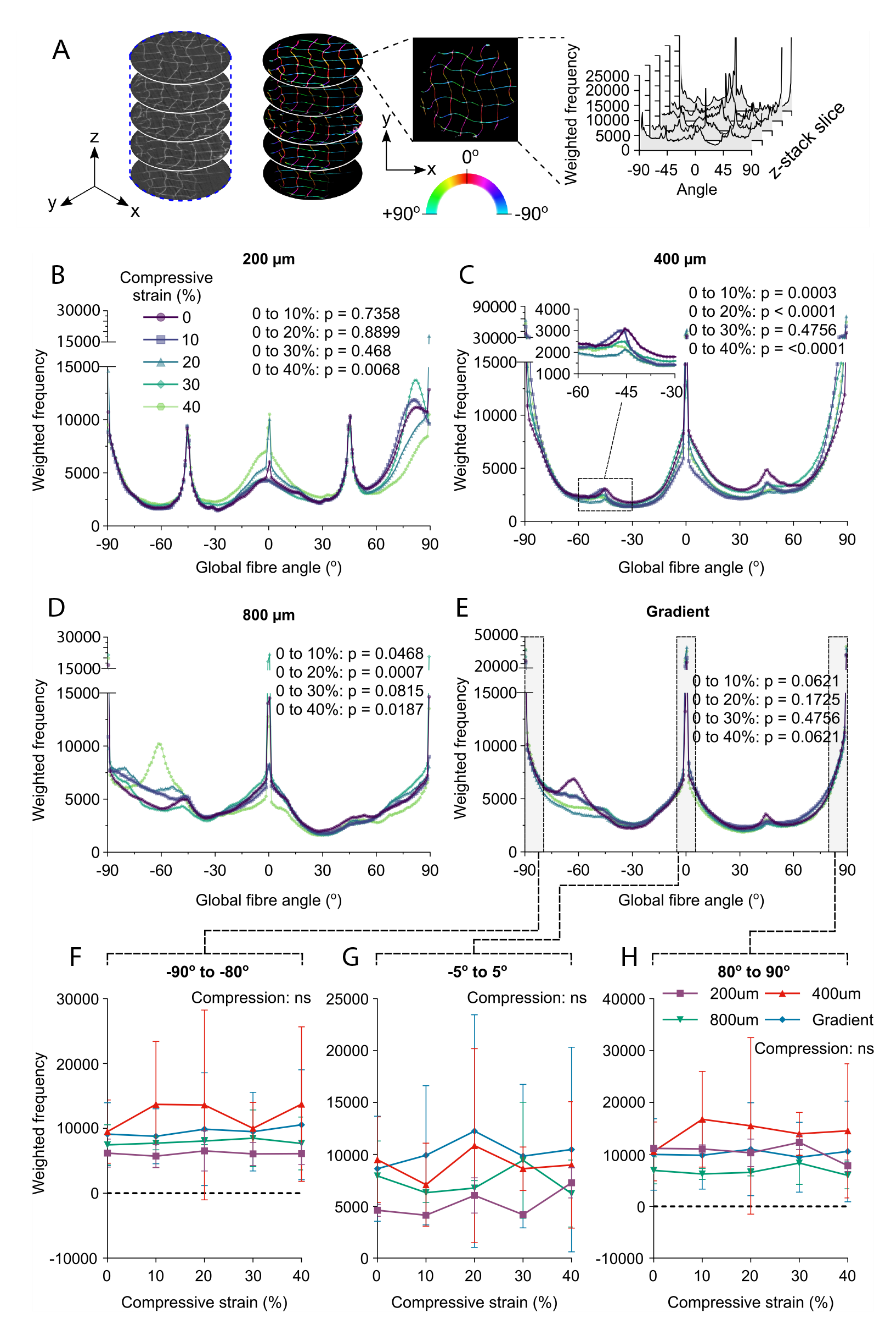


**Figure S4. Fibre orientation of mPCL-reinforced hydrogel constructs at different axial compression steps.** (A) Image slices from synchrotron sCT scans of hydrogel constructs which were analysed using the ImageJ plugin OrientationJ to determine fibre directionality. (B-E) Comparison of global fibre angle weighted histograms of non-deformed and deformed constructs. Statistical significance was determined by comparing weighted histograms at 0% strain with each subsequent 10% strain level (e.g., 10, 20, 30 and 40% strain) using the Kolmogorov-Smirnov test (n = 2 per construct except the 200 µm group which was n = 1). Because scaffolds were printed with a 0-90° lay-down pattern, the differences in weighted fibre angle were quantified at (F) -90 to -80, (G) -5 to 5 and (H) 80 to 90 fibre angles to determine whether fibres were fibres were moving towards or away from the original fibre angle (two-way ANOVA; n = 2 per construct except the 200 µm group which was n = 1).

**DVC analysis of reinforced constructs from sCT in lateral strain directions**


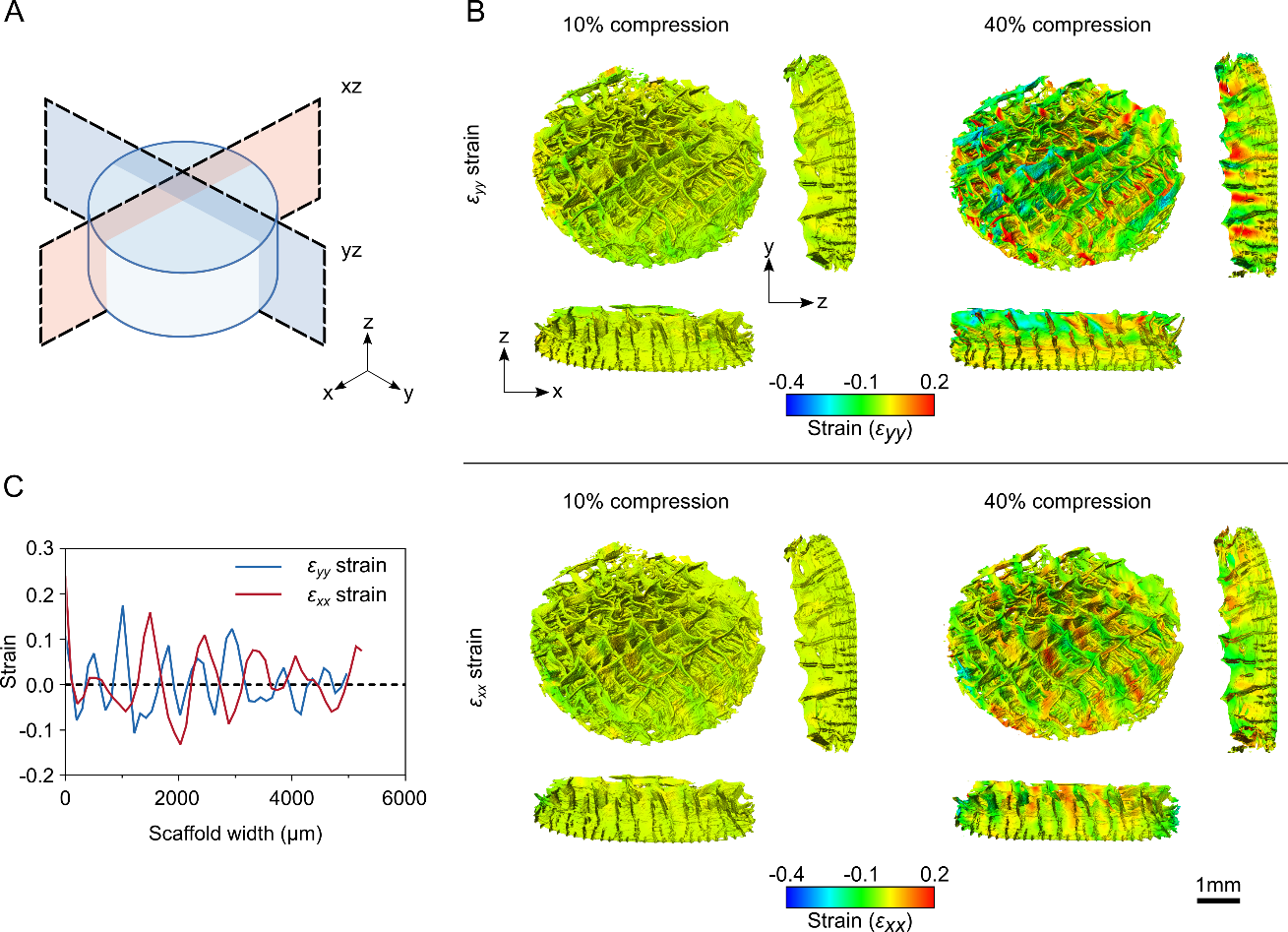


**Figure S5. Lateral (*Ɛ_xx_* and *Ɛ_yy_*) strain DVC analysis of synchrotron x-ray µCT imaged gradient mPCL-reinforced hydrogel constructs at different axial compression steps.** (A) Representative diagram of scaffold showing cut-away plane location for DVC images. (B) 3D reconstructions of constructs with Ɛyy or *Ɛ_xx_* strain superimposed on interior cut-away and angled views of constructs at either 10 or 40% compressive strain. (C) Representative plot of *Ɛ_yy_* and *Ɛ_xx_* strain across the width of the reinforcing scaffold at 40% compression (*n* = 1 construct).

**DVC analysis of gradient constructs from sCT imaging**


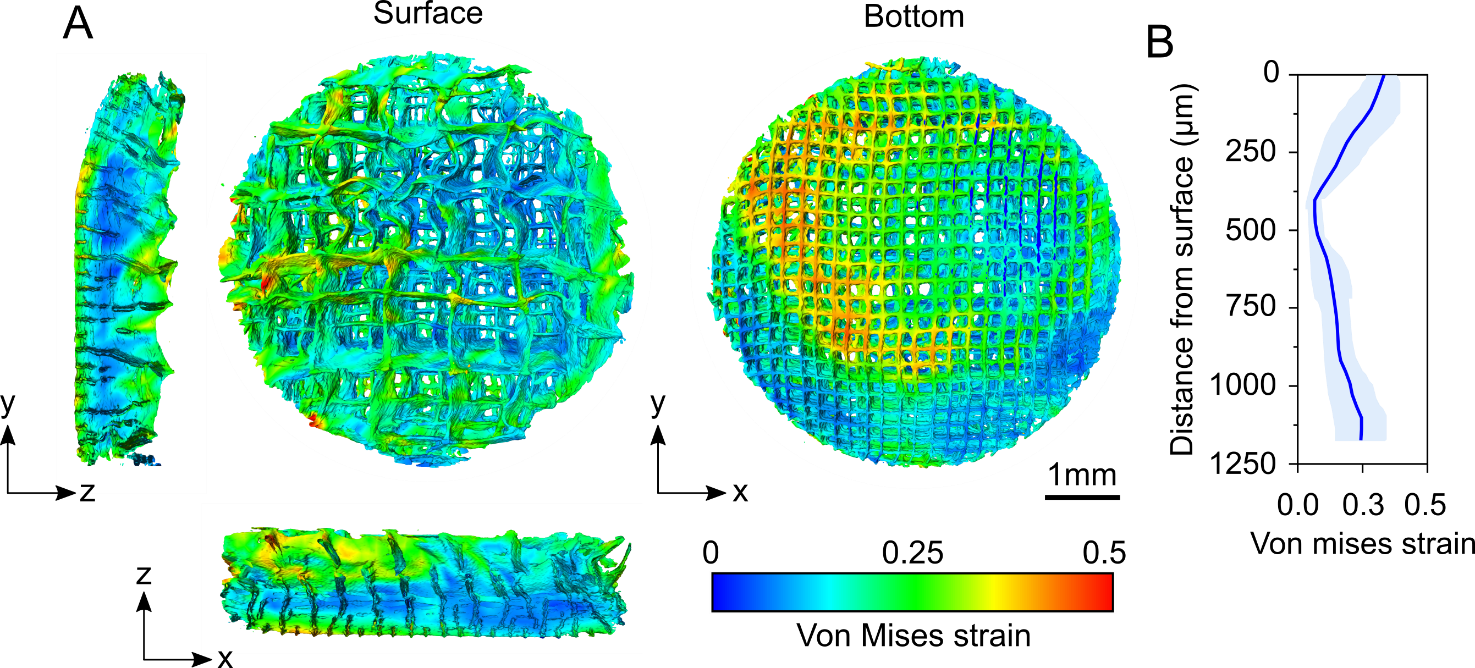


**Figure S6. Equivalent Von Mises strain (Ɛ_eq_) DVC analysis of synchrotron x-ray µCT imaged gradient mPCL-reinforced hydrogel constructs at 40% total compression.** (A) Superimposed Von Mises strain at 40% axial compression of reinforced hydrogel construct side-profile cut-aways, and surface and bottom views of the construct. (B) Plot of Ɛ_eq_ strain across the construct height (mean ± SD; 3 measurements, n = 1 construct).

**FE computational model analysis of gradient constructs**


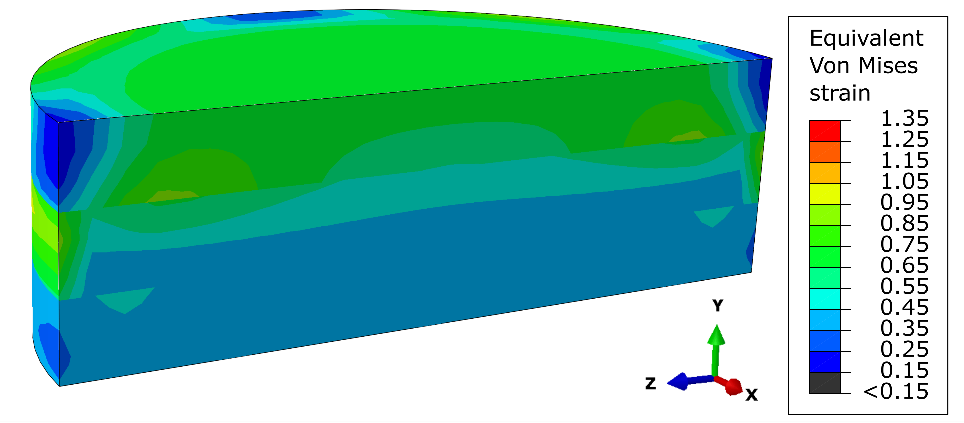


**Figure S7. Equivalent Von Mises strain (Ɛ_eq_) obtained from modelled gradient mPCL-reinforced hydrogel construct at 40% total compression.** The model was otherwise constructed similarly as for optimisations, but here, the used material parameters for each gradient layer were the mean values of the 200 µm, 400 µm and 800 µm group material parameters obtained from the optimisations.

**Parametric FE analysis of mechanical properties of hydrogel and microfibre scaffold**


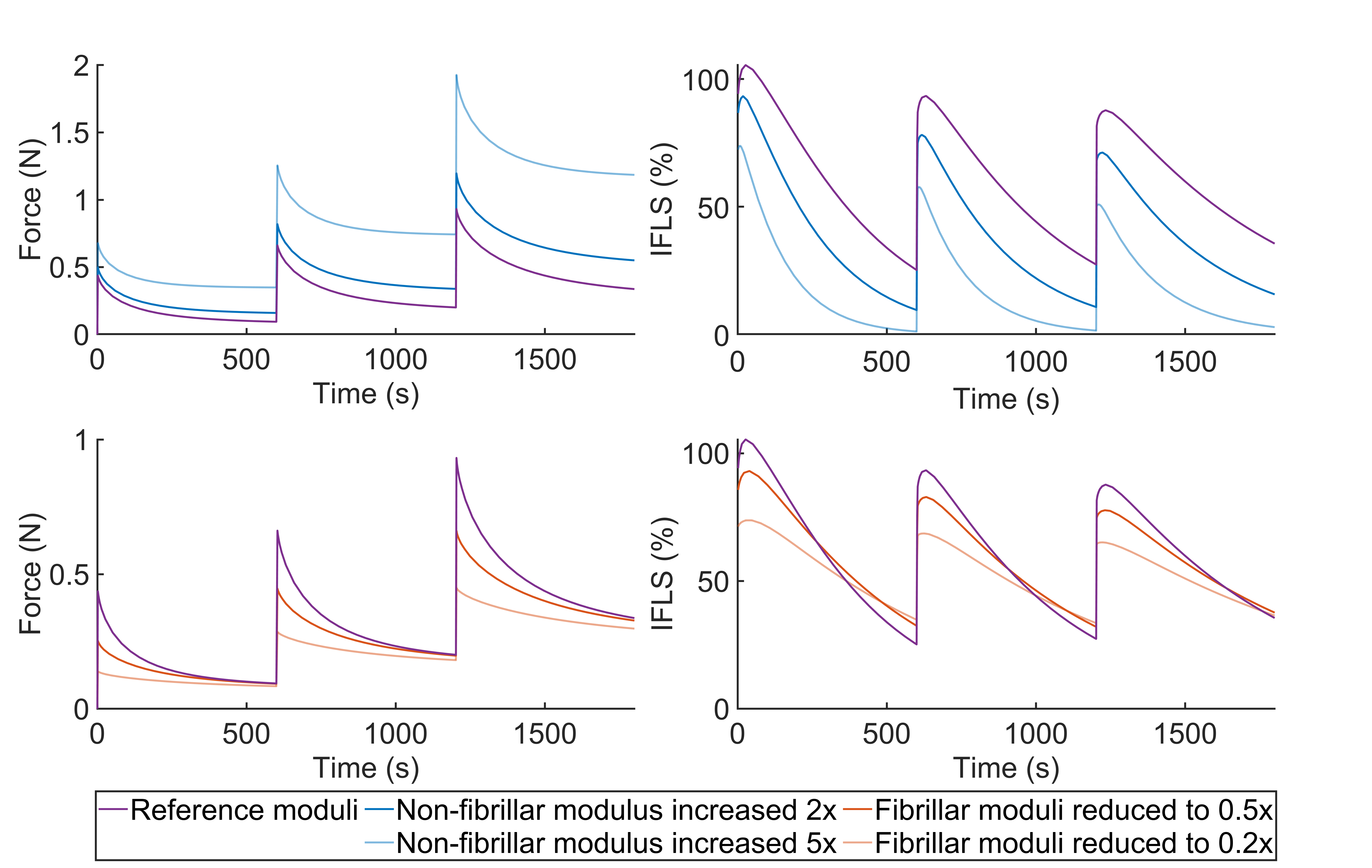


**Figure S8. Parametric evaluation of reaction force (left) and interstitial fluid load support (IFLS) with varying non-fibrillar matrix modulus (top) and initial and strain-dependent fibril network moduli (bottom).**

## Supplementary Tables

| **Supplementary Table 1: The optimised material parameters (mean ± SD) of reinforced and unreinforced hydrogel constructs.** | | | | | |
| --- | --- | --- | --- | --- | --- |
| Reinforced hydrogel constructs | | | | | |
| Group | $E_{f}^{0}$(MPa) | $E_{f}^{\varepsilon}$ (MPa) | $E_{\mathrm{nf}}$ (MPa) | $k_{0}{(10}^{-15}m^{4}N^{-1}s^{-1})$ | $M$ (-) |
| 200 μm (n=5) | 0.014 ± 0.019 | 1.15 ± 0.92 | 0.054 ± 0.005 | 19.0 ± 10.7 | 9.4 ± 2.5 |
| 400 μm (n=6) | 0.015 ± 0.006 | 2.10 ± 0.30 | 0.057 ± 0.009 | 11.6 ± 2.6 | 6.2 ± 0.8 |
| 800 μm (n=6) | 0.007 ± 0.008 | 0.55 ± 0.29 | 0.072 ± 0.013 | 16.6 ± 10.7 | 10.8 ± 4.6 |
| Gradient (n=7) | 0.007 ± 0.008 | 1.08 ± 0.35 | 0.057 ± 0.014 | 16.3 ± 4.1 | 10.0 ± 2.6 |
| Unreinforced hydrogel constructs | | | | | |
| Step | $E_{\text{s}}$ (MPa) | $\bar{g}_{1}$ (-) | $\tau_{1}$(s) | $k_{0}{(10}^{-15}m^{4}N^{-1}s^{-1})$ |  |
| Step 2 | 0.013 ± 0.003 | 0.657 ± 0.062 | 510 ± 143 | 10.191 ± 4.740 |  |
| Step 3 | 0.013 ± 0.003 | 0.785 ± 0.070 | 610 ± 160 | 9.864 ± 6.352 |  |
| Step 4 | 0.013 ± 0.003 | 0.814 ± 0.052 | 976 ± 189 | 10.866 ± 6.836 |  |
| $E_{f}^{0}$ initial fibril network modulus, $E_{f}^{\varepsilon}$ strain-dependent fibril network modulus, $E_{nf}$ non-fibrillar matrix modulus, $k_{0}$ initial permeability, $M$ strain-dependent permeability coefficient, $E_{\text{s}}$ solid matrix modulus, $\bar{g}_{1}$ Prony constant corresponding to dimensionless shear relaxation modulus, $\tau_{1}$ Prony series characteristic relaxation time. | | | | | |

**Supplementary Table 2: FE calculated data for composite samples simulated with 30 min relaxation time per compressive step to obtain simulated equilibrium moduli.**

| Scaffold Group | Simulated equilibrium modulus: Mean [95% confidence interval] (kPa) |
| --- | --- |
| 200 µm | 83 [75 – 91] |
| 400 µm | 89 [75 – 104] |
| 800 µm | 105 [89 – 122] |
| Gradient | 87 [64 – 109] |

## Supplementary References

[1] W. Wilson, C.C. Van Donkelaar, B. Van Rietbergen, K. Ito, R. Huiskes, Stresses in the local collagen network of articular cartilage: a poroviscoelastic fibril-reinforced finite element study, J Biomech 37 (2004) 357–366. https://doi.org/10.1016/S0021-9290(03)00267-7.

[2] A.A. Van der Voet, A comparison of finite element codes for the solution of biphasic poroelastic problems, Proc. Inst. Mech. Eng. H. 211 (1997) 209–211.

[3] W. Wilson, C.C. Van Donkelaar, J.M. Huyghe, A Comparison Between Mechano-Electrochemical and Biphasic Swelling Theories for Soft Hydrated Tissues, J Biomech Eng 127 (2005) 158–165. https://doi.org/10.1115/1.1835361.

[4] P. Tanska, M.S. Venäläinen, A. Erdemir, R.K. Korhonen, A multiscale framework for evaluating three-dimensional cell mechanics in fibril-reinforced poroelastic tissues with anatomical cell distribution – Analysis of chondrocyte deformation behavior in mechanically loaded articular cartilage, J Biomech 101 (2020) 109648. https://doi.org/10.1016/j.jbiomech.2020.109648.

[5] J.T.A. Mäkelä, R.K. Korhonen, Highly nonlinear stress-relaxation response of articular cartilage in indentation: Importance of collagen nonlinearity, J Biomech 49 (2016) 1734–1741. https://doi.org/10.1016/j.jbiomech.2016.04.002.

[6] L.P. Li, J. Soulhat, M.D. Buschmann, A. Shirazi-Adl, Nonlinear analysis of cartilage in unconfined ramp compression using a fibril reinforced poroelastic model, Clinical Biomechanics 14 (1999) 673–682. https://doi.org/10.1016/S0268-0033(99)00013-3.

[7] J.-K.K. Suh, M.R. Disilvestro, Biphasic poroviscoelastic behavior of hydrated biological soft tissue, Journal of Applied Mechanics, Transactions ASME 66 (1999) 528–535. https://doi.org/10.1115/1.2791079.

[8] S. Kalyanam, R.D. Yapp, M.F. Insana, Poro-viscoelastic behavior of gelatin hydrogels under compression- implications for bioelasticity imaging, J Biomech Eng 131 (2009) 1–13. https://doi.org/10.1115/1.3127250.

[9] J.C. Lagarias, J.A. Reeds, M.H. Wright, P.E. Wright, Convergence properties of the Nelder-Mead simplex method in low dimensions, SIAM Journal on Optimization 9 (1998) 112–147. https://doi.org/10.1137/S1052623496303470.

[10] O. Bas, E.M. De-Juan-Pardo, M.P. Chhaya, F.M. Wunner, J.E. Jeon, T.J. Klein, D.W. Hutmacher, Enhancing structural integrity of hydrogels by using highly organised melt electrospun fibre constructs, Eur Polym J 72 (2015) 451–463. https://doi.org/http://dx.doi.org/10.1016/j.eurpolymj.2015.07.034.
